# Effects of different art therapies on the psychological well-being and quality of life in cancer survivors: a systematic review and network meta-analysis

**DOI:** 10.3389/fpsyt.2026.1817092

**Published:** 2026-07-06

**Authors:** Qianlu Wang, Yue Tao, Ying Huang, Bihong Huang

**Affiliations:** Nursing Department, Huashan Hospital Affiliated to Fudan University, Shanghai, China

**Keywords:** art therapy, cancer survivors, network meta-analysis, psychological well-being, quality of life

## Abstract

**Objective:**

Art therapy has gained increasing attention as an adjunctive intervention for cancer patients. This study sought to conduct the first network meta-analysis (NMA) to evaluate the effects of various art therapies on the psychological well-being and quality of life in cancer survivors, rank various interventions by efficacy, and provide evidence-based guidance for clinical practice.

**Methods:**

Web of Science, PubMed, Embase, and the Cochrane Library, were systematically searched for studies published up to September 3, 2025. Eligible studies were selected according to predefined criteria, and data were independently extracted by two researchers. Heterogeneity was assessed using the I² statistic, and fixed- or random-effects models were applied accordingly. A Bayesian NMA was conducted to compare interventions and rank their efficacy using the surface under the cumulative ranking curve (SUCRA). Publication bias was assessed using comparison-adjusted funnel plots and Egger’s test. Network meta-regression was performed for outcomes with high heterogeneity. Analyses were conducted using R Studio v4.5.2 and STATA v15.0.

**Results:**

Twenty-eight studies involving 2,542 cancer survivors were included. The NMA demonstrated that mandala painting therapy exhibited statistically significant efficacy in alleviating depression and enhancing quality of life. Regarding fatigue reduction, music therapy was superior to routine care (standardized mean difference [SMD] = -5.83, 95% credible interval [CrI]: -6.65, -5.02) and had the highest SUCRA ranking (96.4%). In terms of anxiety and pain relief, although music therapy had high SUCRA rankings (67.4% and 69.0%, respectively), pairwise comparisons did not show statistically significant differences.

**Conclusion:**

Mandala art therapy and music therapy may improve psychological well-being and quality of life among cancer survivors. However, because of the absence of closed loops in the evidence network, substantial clinical heterogeneity, and potential publication bias, SUCRA-based rankings should be interpreted cautiously. Further high-quality, multi-arm, standardized randomized controlled trials with lower risk of bias are needed to confirm these findings.

**Systematic Review Registration:**

https://www.crd.york.ac.uk/PROSPERO/, identifier CRD420251149102

## 1 Introduction

With continuous advances in medical technology, the survival rates of cancer patients have improved significantly, and the global number of cancer survivors continues to grow (1, 2). The U.S. National Comprehensive Cancer Network (NCCN) defines a cancer survivor as an individual from the moment of diagnosis until the end of life (2). However, increased survival rates have also brought new challenges. The diagnosis of cancer and its subsequent treatment not only cause physical suffering to the patients, but may also induce or exacerbate mental health problems, and may even lead to suicidal thoughts (3). The resulting psychological distress is not merely a temporary emotional fluctuation, but is often persistent and can severely impair patients’ quality of life and overall well-being.

Among these, anxiety and depression are particularly common and prominent problems. A meta-analysis (4) has shown that the combined prevalence of anxiety and depression among cancer patients worldwide remains high, with the prevalence of depression in breast cancer patients reaching as high as 68% (5). Existing research (6) has confirmed that psychological distress is significantly negatively correlated with all dimensions of quality of life and remains prevalent for up to 25 years after diagnosis (7). It has gradually become a public health issue that urgently requires attention and intervention. Given its profound impact on patient prognosis, symptom management has been established as a core component of cancer care by authoritative international guidelines. In 2023, the Society for Integrative Oncology (SIO) and the American Society of Clinical Oncology (ASCO) jointly published the *Integrative Oncology Care of Symptoms of Anxiety and Depression in Adults With Cancer*. NCCN and the European Society for Medical Oncology (ESMO) have also emphasized the need for systematic screening and intervention for such psychosocial issues. This study selected anxiety and depression as core outcome measures not only because they are commonly reported psychological burdens among cancer survivors but also because they have well-established quantification standards, facilitating the pooling and comparison of standardized mean differences (SMD) across different art interventions. Furthermore, considering the interaction between psychological and physiological functions, this study concurrently included key outcome measures, such as pain, fatigue, and quality of life, to provide a multidimensional assessment of the effectiveness of art therapy for cancer survivors.

In the field of psychological intervention, art therapy has garnered significant attention in recent years. First proposed by Adrian Hill in the 1940s (8), this therapy uses artistic media to facilitate emotional expression and psychological healing and assists patients in coping with somatic symptoms and complex emotions (9). Current evidence (10, 11) indicates that art therapy plays a positive role in improving the mental health of cancer patients and alleviating the burden of symptoms. Art therapy encompasses a diverse range of modalities (11), including music, dance/movement, visual arts, and narrative creation. To explain the psychological mechanisms underlying different art forms, the Expressive Therapies Continuum (ETC) (12) posits that various art forms can facilitate emotional regulation and psychological integration by activating different levels of psychological processing, ranging from kinesthetic perception to cognitive symbols. Specifically, music therapy engages basic sensory perception through the auditory pathway, facilitates emotional regulation, and alleviates physical symptoms. It is currently one of the intervention forms with the most robust evidence base (13, 14). Some culturally specific musical forms, such as Sufi music centered on the Ney flute, have been shown to significantly improve mental health outcomes in cancer patients due to their meditative and calming qualities (15). Similarly, dance/movement therapy promotes emotional release through physical movement and rhythmic experience (16, 17). Visual arts therapy primarily works at the affective-perceptual level; for instance, mandala painting externalizes implicit emotions into structured images to alleviate anxiety (18). Self-book therapy focuses on cognitive integration and guides patients to symbolically integrate fragmented emotions to enhance psychological resilience and self-identity (19, 20).

Although art therapy has demonstrated some positive effects, the existing evidence remains limited. Reports on efficacy vary across studies (21–23). More critically, the vast majority of randomized controlled trials (RCTs) have compared only a single form of art intervention with routine care or a blank control, and the relative efficacy of various art therapies remains unclear. To address this evidence gap, this systematic review and network meta-analysis (NMA) was conducted to comprehensively evaluate the intervention effectiveness of different art therapies among cancer survivors by synthesizing direct and indirect evidence. We ranked the interventions by their relative efficacy for outcome measures including anxiety, depression, quality of life, pain, and fatigue, aiming to provide evidence-based guidance for clinical practice.

## 2 Methods

This study was conducted following the Preferred Reporting Items for Systematic Reviews and Meta-Analyses Extension for Network Meta-Analyses (PRISMA-NMA) guidelines (24) and the protocol registered in the International Prospective Register of Systematic Reviews (PROSPERO) (CRD420251149102), which predefined the research plan for this review.

### 2.1 Literature search

A comprehensive computerized search was conducted across the Web of Science, PubMed, Embase, and Cochrane Library databases for RCTs examining the effects of various art therapies on the psychological well-being and quality of life in cancer survivors. The search covered articles published up to September 3, 2025. Search terms included “neoplasms”, “art”, “music”, and “painting”. Only articles written in English were retrieved. MeSH terms and free-text words were combined utilizing Boolean operators (AND, OR) to develop the search strategy, which was tailored to each database (**Supplementary Table 1**). Additionally, reference lists of included studies were also searched to obtain additional data and avoid omissions.

### 2.2 Screening criteria

#### 2.2.1 Inclusion criteria

Based on the PICOS search algorithm, inclusion criteria were established as follows (1): Participants: adult cancer survivors (defined as per NCCN guidelines) (2); Intervention: various art therapies (including music, dance, painting, etc.) (3); Comparison: routine care or any interventions that did not involve artistic media, such as health education or relaxation training (4); Outcomes: outcome variables included anxiety, depression, fatigue, pain, and quality of life. All included studies must report at least one of the above outcomes. The five outcome measures were pre-specified during the study design phase and were carefully selected with full consideration of clinical needs (5); Study design: RCT.

#### 2.2.2 Exclusion criteria

Exclusion criteria comprised (1): Observational studies, letters, reviews, case reports, conference abstracts, letters to the editor, or editorials (2); Animal studies (3); Articles with incomplete or unavailable data (4); Articles for which the full text could not be retrieved (5); Articles reporting no relevant outcome measures (6); Articles with study designs that did not meet the prespecified requirements.

### 2.3 Literature screening

Two researchers (WQL and TY) independently screened the retrieved articles based on the predefined inclusion and exclusion criteria. First, duplicate articles were removed using EndNote 21. The remaining articles were then preliminarily reviewed by title and abstract review. Articles that could not be definitively excluded during the preliminary screening were included for full-text assessment. The two researchers independently re-screened the downloaded full texts to determine final eligibility for inclusion in this study. In the event of any disagreement at any screening stage, a third researcher (HY) was consulted, and the final decision was reached through discussion until consensus was reached to ensure objectivity and consistency in the screening process.

### 2.4 Data extraction

Two researchers (WQL and TY) independently screened and re-checked the articles according to the predefined inclusion and exclusion criteria. Subsequently, data were extracted from the included studies, including first author, publication year, region, sample size, grouping, age and gender distribution of participants, type of cancer, clinical staging, baseline treatment regimens, interventions and controls, and the outcome measurement tools in each study. All extracted data were cross-checked by two researchers. Discrepancies, if any, were addressed through discussion with a third researcher (HY) until consensus was reached, so as to ensure consistency and accuracy in data extraction.

### 2.5 Quality assessment

Two researchers (WQL and TY) independently assessed the quality of included studies using the Cochrane risk of bias tool version 2.0 (ROB 2.0). The assessment covered five core domains (1): randomization process (2); deviations from intended interventions (3); missing outcome data (4); outcome measurement (5); selection of the reported result. Based on these five domains, the overall risk of bias for each study was further assessed and categorized into three levels: low risk of bias, some concerns, or high risk of bias. After completing independent assessments, the two researchers cross-checked the assessment results. In case of discrepancies, a third researcher (HY) participated in discussions until consensus was reached to ensure consistency and reliability of the assessment.

### 2.6 Statistical analysis

This study employed a Bayesian NMA utilizing Stata 15.0 and the gemtc package in R Studio 4.5.2 (25, 26). Continuous variables were presented as standardized mean differences (SMD) and their 95% credible intervals (CrI). The Markov chain Monte Carlo (MCMC) method was used for model construction, with 4 chains, 20,000 iterations, and a burn-in period of 5,000 iterations. Model convergence was assessed using a combination of the Brooks-Gelman-Rubin potential scale reduction factor (PSRF) and trace plots. Heterogeneity was assessed using the I^2^ statistic (27): if I^2^ > 50%, indicating high heterogeneity, a random-effects model was used; if I^2^ < 50%, indicating no significant heterogeneity, a fixed-effects model was used (28). For outcome measures with high heterogeneity (I^2^ > 50%), univariate meta-regression analyses were further conducted, using publication year, sample size, and gender ratio as covariates, to explore potential sources of heterogeneity. The criterion for determining whether a covariate has a significant moderating effect was whether the 95% CrI of the regression coefficient (B) contained 0. If a closed loop existed in the evidence network, the node-splitting method was used to assess the consistency between direct and indirect evidence. If the evidence network exhibited a star-shaped distribution and did not contain a closed loop, consistency model analysis was primarily based on the transitivity assumption. The relative efficacy of the interventions was presented in league tables, and the surface under the cumulative ranking curve (SUCRA) values were calculated to predict the probability ranking of the art therapies. A higher SUCRA value indicated a greater probability of the therapy being the most effective. Statistical inference for outcome measures was based on the posterior distribution. If the 95% CrI of the SMD did not contain the null value (0), the intervention effectiveness was considered statistically significant. Comparison-adjusted funnel plots combined with Egger’s test were used to quantitatively assess potential publication bias or small-sample effects. All ancillary analyses were conducted using two-sided tests, with P < 0.05 (29) defined as statistical significance. As a systematic review, this study did not involve the recruitment of or participation by patients or the public.

### 2.7 Sensitivity analysis

Given that some of the included studies did not fully report cancer staging, baseline treatment, or key characteristics of participants, which may increase clinical heterogeneity among studies and the risk of selection bias, targeted sensitivity analyses were further conducted. After excluding studies reporting incomplete data, an NMA was performed on the primary outcome measures to assess the stability of the effect direction, effect size, and SUCRA ranking results. If the results did not change substantially before and after exclusion, the results of the main analysis were considered to be robust.

## 3 Results

### 3.1 Literature screening results

**Figure 1:**
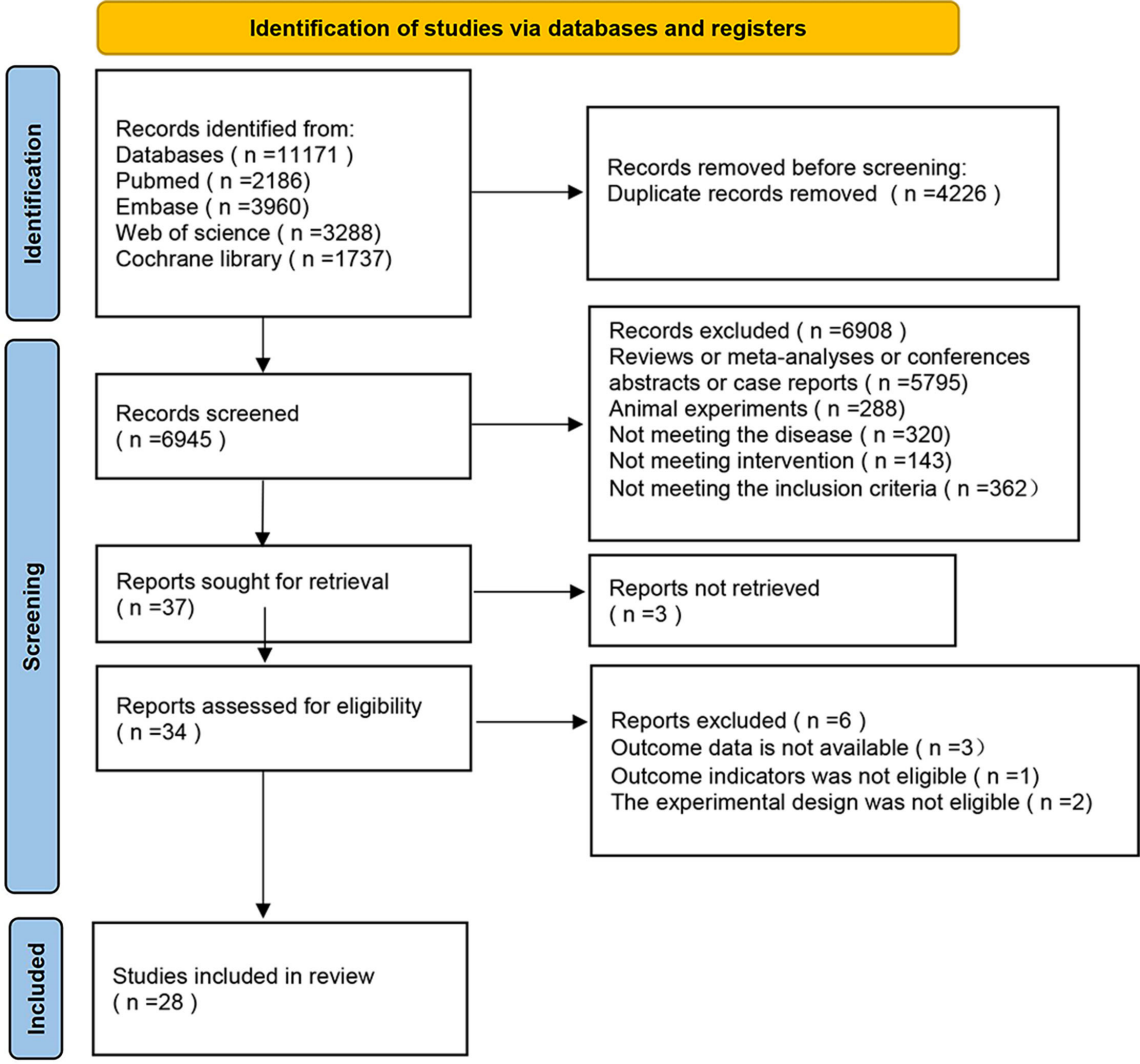
Literature screening process (30).

The initial search yielded 11,171 articles. After removing 4,226 duplicates and excluding 6,908 articles based on title and abstract screening, 3 articles with no available full texts and 6 ineligible articles were further excluded. Ultimately, 28 articles were included. The detailed screening process is shown in **Figure 1**.

### 3.2 Characteristics of included studies

The 28 included studies (22, 23, 31–56) involved 2,542 patients in total, including 670 males (26.36%), 1,870 females (73.56%), and 2 with other genders (0.08%). The included studies spanned from 2003 to 2025. Among them, 20 involved music therapy, 3 involved mandala painting therapy, 4 involved dance therapy, and 1 involved self-book therapy. The mean age range of patients was 43.5–66 years for the T (treatment) groups and 41.1–65 years for the C (control) groups. Overall, among the 2,542 patients included in the studies, breast cancer was the most common type of disease, followed by lung cancer, gastrointestinal tumors, hematological malignancies, and prostate cancer. Some studies did not specify the baseline treatment, cancer staging, or specific type of cancer, merely describing them as other cancers or advanced cancers. Additional information is presented in **Table 1**.

**Table 1:** Summary of included studies.

Study	Region	Participants (T/C)	Age(Mean ± SD)	Gender (M/F/other)	Cancer staging	Disease type	Baseline treatment	Treatment group	Control group	Evaluation tool	Provider	(Duration & frequency)
Havva2024	Turkey	60(30/30)	T:60.13 ± 10.77; C:61.07 ± 13.78	T;15/15 C:18/12	NA	Lung, cancer, Breast cancer, GI cancer, Other cancers	chemotherapy	Music therapy	routine care	BFI, STAI	professionals	30 mins, 3 sessions
Felicity2023	American	708(376/332)	T:60.26 ± 12.43; C:60.54 ± 12.59	T;135/240/1 C:112/220	Stage0–IV	NA	chemotherapy	Music therapy	routine care	VAS, PANAS, Distress Thermometer	professionals	60 min, 1 session
Francesco2014	Italy	52(26/26)	T:64.3 ± 12.9; C:64.6 ± 12.8	T:1/25 C:8/18	StageI-IV	NA	Pain relief	Music therapy	routine care	VAS-P, VAS-M	professionals	30 mins, 4 sessions
Barrie2003	American	69(36/33)	T:53.0 ± 11.0; C:51.0 ± 12.0	T:22/14; C:10/23	Hematologic	Lymphoma, Multiple myeloma, Amyloidosis	High-dose therapy, Autologous stem cell transplantation	Music therapy	routine care	POMS	therapists	30 mins, 5 sessions
Shu2019	TW,China	94(46/48)	T:51.16 ± 9.21; C:49.56 ± 9.78	female-only	Stage0–IV	Breast Cancer	chemotherap, mastectomy	Music therapy	routine care	MFSI-SF, HADS	therapists	45 mins, 1 session
Parvaneh2024	Iran	70(35/35)	GroupA:45.51 ± 9.32; GroupB:44.94 ± 9.98	female-only	NA	Breast Cancer	chemotherapy	Mandala(Group A)	Sudoku (Group B)	STAI	professionals	120 mins, 1 session
Ching2024	TW,China	128(63/65)	T:52.0 ± 10.0; C:53.2 ± 9.6	female-only	Stage0–IV	Breast Cancer	mastectomy, chemotherapy, Radiation therapy	Music therapy	routine care	WHOQOL-BREF, STAI, BFI	professionals	60 mins, 12 sessions
Suzanne2014	American	59(29/30)	T:50.2 ± 14.1; C:51.6 ± 13.8	T:10/19; C:14/16	Hematologic	Leukemia, Lymphoma, myeloma	local anesthesia for Bone Marrow Biopsy	Music therapy	routine care	STAI, VAS	professionals	5-4 0mins, 1 session
Russell2003	American	80(40/40)	T:66 ± 12.2 C:65 ± 12.2	T:20/20; C:20/20	Stage IV	advanced-stage cancer	Routine hospice services	Music therapy	routine care	HQOLI-R	therapists	30–45 mins, 2 sessions
Rainbow2016	HK,China	139(69/70)	T:48.6 ± 7.7; C:49.1 ± 8.7	female-only	Stage0–III	Breast Cancer	mastectomy, chemotherapy, Radiation therapy	Dance therapy	routine care	HADS-A, HADS-D, BFI	therapists	90 mins, 6 sessions
Talita2020	Brazil	33(16/17)	T:49.50 ± 10.65;C: 50.76 ± 9.45	female-only	StageI-II	Breast Cancer	chemotherapy	Music therapy	routine care	WHOQOL, BAI	professionals	30 mins, 3 sessions
Mei2010	TW,China	98(34/30/34)	Total:53.0 ± 12.5	Group A:13/21; Group B:9/21; Group C:11/23	StageI-IV	Breast cancer, lung cancer and other cancers	chemotherapy	Group A:Music therapy Group B:Verbal meditation	routine care	STAI	professionals	60 mins, 1 session
Madison2021	American	44(24/20)	T:57.04 ± 12.13; C:52.85 ± 16.00	T:(6/18);C:(5/14/1)	NA	Colon cancer, stomach cancer, and other cancers	surgery	Music therapy	routine care	GMS, VAS	therapists	30 mins, 1 session
KIRTISHRI 2022	American	40(20/20)	T: 64.88 ± 6.23; C:62.13 ± 7.50	Male- only	NA	prostatic cancer	surgery	Music therapy	routine care	STAI	professionals	30 mins, 2 sessions
Murat2023	Turkey	60(30/30)	Frequency&Percentage T:26-35y:**3(10%)**;36-45y:**12(40%)**;46-65y:**11(36.7%)**;≥65y:**4(13.3%)** C:26-35y:**6(20%)**;36-45y:**5(16.7%)**;46-65y:**13(43.3%)**;≥65y:**6(20%)**	female-only	StageI-III	Breast cancer	surgery, chemotherapy	Ney Music therapy	routine care	VAS, BAI, FACT-G	professionals	30 mins, 5 sessions
Clare2012	Australian	100(50/50)	T:57 ± 14.2; C:58 ± 12.7	T:30/20; C:29/21	NA	prostatic cancer, Breast cancer and other cancers	radiotherapy	Music therapy	routine care	STAI	Support Staff	30 mins. 1 session
Friedeman2023	German	52(24/28)	T:60.5 ± 10.3; C:59.3 ± 11	female-only	StageI-III	Breast Cancer	surgery, chemotherapy, Radiation therapy	Dance therapy	Routine care	CFS-D, EORTC QLQ-C30	professional	60 mins, 6 sessions
Donna2018	American	40(20/20)	T:51.95 ± 10.59; C:52.30 ± 12.42	female-only	StageI-IV	Breast cancer, lung cancer and other cancers	chemotherapy, Radiation therapy	self book	Routine care	DT, PROMIS	therapists	50 mins, 6 sessions
Tal2023	Israel	30(14/16)	Total:54.3 ± 11.5	Total:2/28	NA	Breast Cancer and other cancers	chemotherapy, bone marrow transplantation, biotherapy	Music therapy	Routine care	STAI, VAS	therapists	60 mins, 1 session
Rajeev2025	India	44(22/22)	T:49.9 ± 13.8; C:49.1 ± 17.1	T:12/10; C:12/10	NA	GI cancer and other cancers	surgery	Music therapy	routine care	VAS, DASS-21	professionals	30 mins. 14 sessions
Huei2024	TW,China	100(50/50)	T:59.9 ± 9.6 C:60.3 ± 11.3	T:25/25; C:23/27	StageI-IV	Breast Cancer and other cancers	surgery, chemotherapy, Radiation therapy	Music therapy	routine care	BAI, DT	Support Staff	10–15 mins. 140sessions
Yu2025	TW,China	66(33/33)	Total:53.2 ± 10.3	female-only	Stage0–IV	Breast Cancer	Surgery, Chemotherapy, Radiotherapy, Targeted therapy	Dance therapy	routine care	EORTC QLQ-C30	professionals	60 mins, 12 sessions
Kemik2025	Turkey	46(23/23)	NA	T:12/11; C:11/12	Hematologic	Lymphoma, Multiple myeloma and other cancers	chemotherapy	Mandala	routine care	DT, STAI	professionals	30 mins, 7 sessions
Pamela2011	American	30(15/15)	Total:56.63	female-only	NA	Breast Cancer	Surgery	Music therapy	routine care	STAI	professionals	Perioperative, 1 session
Nasrin2025	Iran	70(35/35)	T:43.45 ± 8.03; C:44.00 ± 8.20	female-only	StageI-III	Breast Cancer	surgery, chemotherapy, Radiation therapy	Mandala	routine care	EORTC QLQ-C30	professionals	45 mins, 6 sessions
Marta2015	Hungary	114(55/59)	T:48.87 ± 8.88 C:51.31 ± 11.06	female-only	NA	Breast Cancer and other cancers	Chemotherapy, Hormone therapy,	Dance therapy	routine care	EORTC QLQ-C30	professionals	180 mins, 52 sessions
Maryam2023	Iran	54(25/29)	T:47.74 ± 13.96; C:41.14 ± 9.87	T:4/21; C:4/25	NA	Breast cancer, lung cancer and other cancers	Surgery, chemotherapy, Radiation therapy	Music therapy	routine care	STAI, VAS	professionals	20 mins, 16 sessions
Ozgur2023	Turkey	62(32/30)	T:51.80 ± 3.62; C:53.35 ± 3.18	T:19/13; C:18/12	StageII-III	Colon cancer and Rectal adenocarcinoma	chemotherapy	Music therapy	routine care	STAI, BDI	professionals	45 mins, 6 sessions

### 3.3 Quality assessment

**Figure 2:**
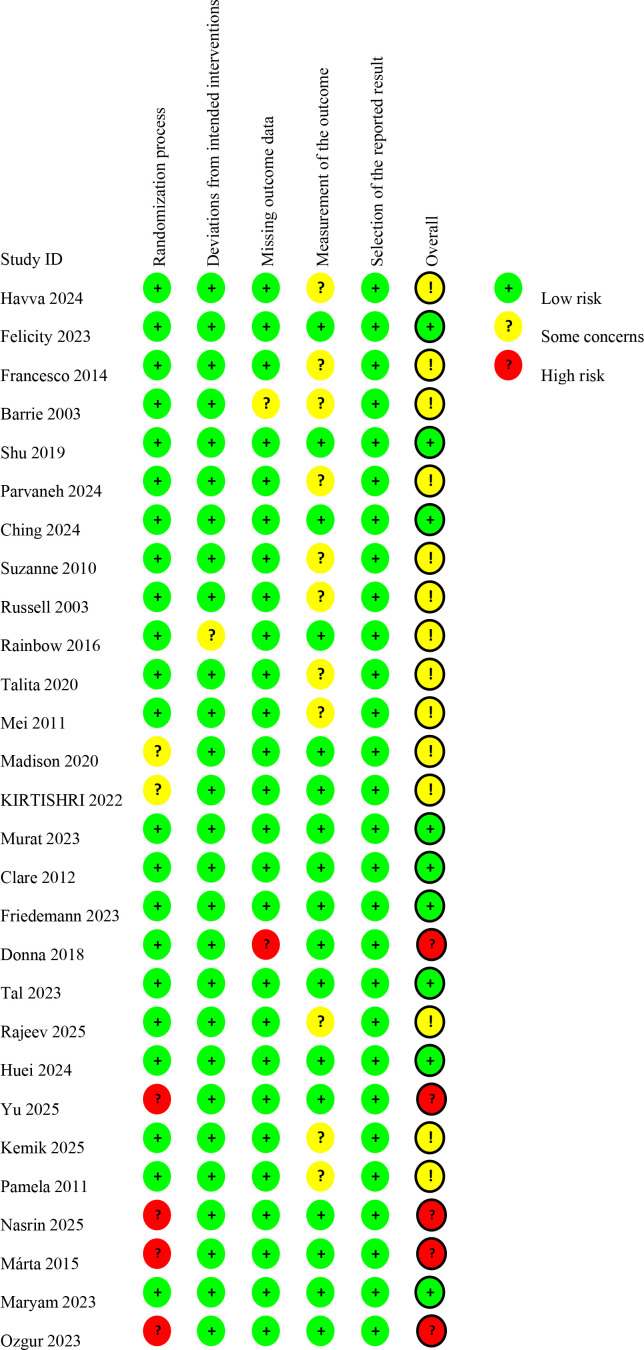
Risk of bias assessment results.

The quality of the 28 studies included was assessed utilizing the Cochrane ROB 2.0 tool. Among them, 9 studies were rated as overall low risk of bias, while 14 studies were rated as some concerns because they were rated as some concerns in at least 1 domain. Of the remaining 5 studies, 4 were rated as high risk primarily due to failure to clearly report the true randomization method or a lack of adequate measures for allocation concealment, and 1 was rated as high risk due to a 33% loss to follow-up rate and the absence of sensitivity analysis for processing missing data. The quality assessment results are depicted in **Figure 2**.

### 3.4 Network diagrams

**Figure 3:**
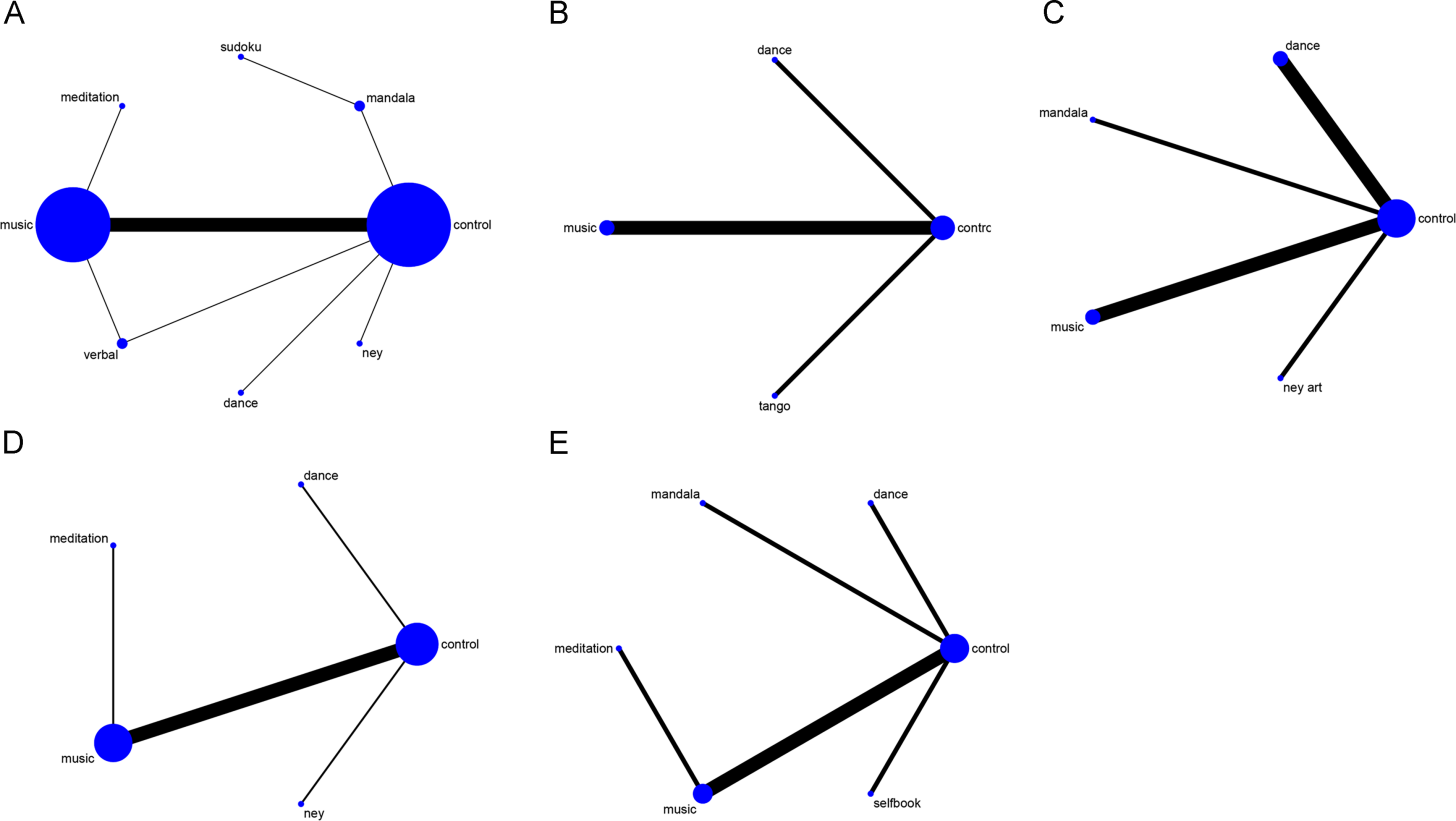
Comparison of different art therapies versus routine care for efficacy in outcome measures among cancer survivors: anxiety **(A)**, fatigue **(B)**, quality of life **(C)**, pain **(D)**, depression **(E)**.

In the network diagrams, the nodes represented different art therapies included. Node size was proportional to the total sample size for each intervention, with larger nodes indicating greater cumulative sample size for that intervention. Solid lines between nodes indicated direct comparisons between two interventions in the original studies. Line thickness correlated positively with the number of studies involving the direct comparison between the two interventions, with thicker lines indicating a greater number of studies involving the pairwise comparison (**Figure 3**).

### 3.5 NMA

#### 3.5.1 Anxiety

**Figure 4:**
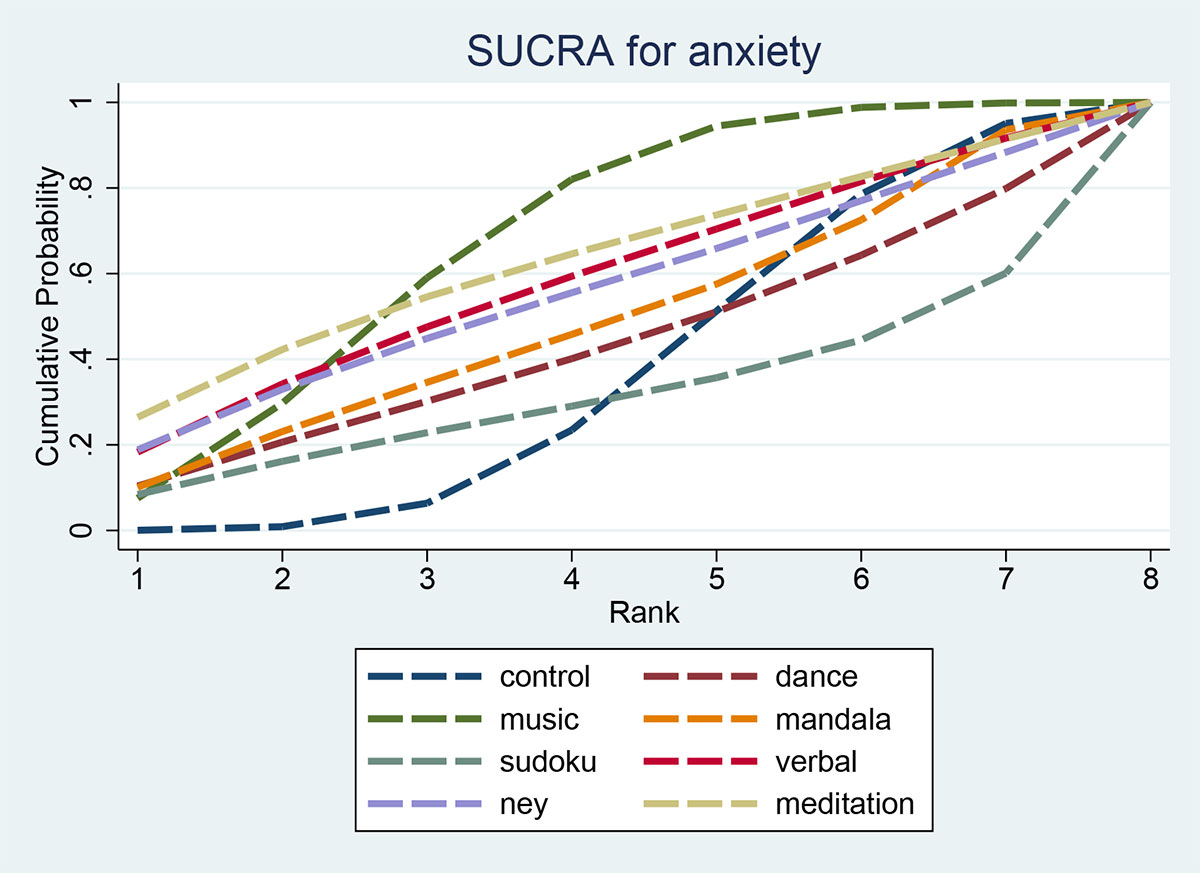
Area under the cumulative ranking curve for anxiety.

The league table indicated no statistically significant differences between the interventions and control in the reduction of anxiety. Additional data are presented in **Table 2**. The interventions were ranked by SUCRA value as follows: music therapy (67.4%) > meditation (62.3%) > speech therapy (57.6%) > Ney therapy (54.8%) > mandala painting therapy (48.2%) > dance therapy (42.4%) > routine care (36.5%) > Sudoku therapy (31.0%). The SUCRA ranking results suggested that music therapy (67.4%) was likely to rank high. However, since the league table showed no statistically significant differences among the interventions, this ranking should be considered only as exploratory and not as definitive evidence of superiority (**Figure 4**).

**Table 2:** League table for anxiety.

**control**							
-0.05 (-35.33, 35.08)	**dance**						
9.23 (-0.21, 18.8)	9.22 (-27.16, 46.01)	**music**					
2.27 (-33.04, 37.23)	2.12 (-47.64, 52.02)	-7.02 (-43.78, 29.16)	**mandala**				
-7.57 (-57.69, 42.27)	-7.58 (-68.12, 53.65)	-16.79 (-67.95, 33.9)	-9.79 (-45.34, 25.73)	**sudoku**			
7.18 (-23.93, 38.75)	7.31 (-39.29, 54.75)	-2.04 (-33.15, 29.47)	5.03 (-41.98, 52.28)	14.85 (-43.66, 74.14)	**verbal**		
6.03 (-29.52, 41.19)	6.05 (-43.94, 56.2)	-3.21 (-39.79, 33.14)	3.81 (-45.9, 53.55)	13.58 (-47.36, 74.47)	-1.22 (-48.56, 46.21)	**ney**	
9.84 (-26.58, 46.1)	9.71 (-40.11, 60.59)	0.58 (-34.69, 35.63)	7.63 (-43.26, 58.47)	17.45 (-44.38, 79.67)	2.66 (-44.41, 49.13)	3.89 (-46.92, 54.18)	**meditation**

#### 3.5.2 Fatigue

**Figure 5:**
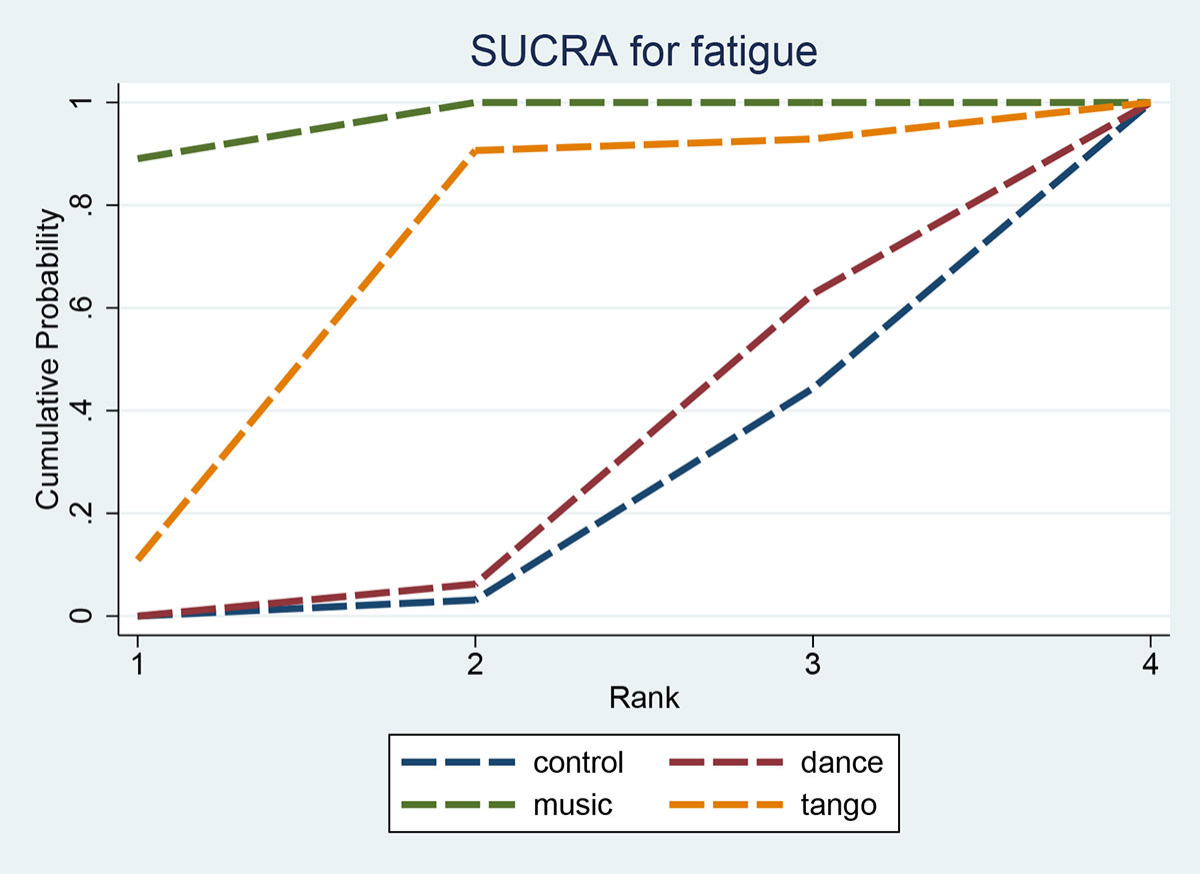
Area under the cumulative ranking curve for fatigue.

The league table indicated that music therapy outperformed dance therapy (SMD = -5.73, 95% CrI: -4.63, -6.84) and routine care (SMD = -5.83, 95% CrI: -5.02, -6.65) in reducing fatigue. Additional data are presented in **Table 3**. The interventions were ranked by SUCRA value as follows: music therapy (96.4%) > Tango therapy (64.8%) > dance therapy (23.0%) > routine care (15.8%). This suggested that music therapy may offer certain benefits in alleviating fatigue among cancer survivors (**Figure 5**).

**Table 3:** League table for fatigue.

**control**			
0.1 (-0.65, 0.85)	**dance**		
5.83 (5.02, 6.65)	5.73 (4.63, 6.84)	**music**	
3.09 (-1.14, 7.36)	2.99 (-1.3, 7.33)	-2.74 (-7.06, 1.63)	**tango**

#### 3.5.3 Quality of Life

**Figure 6:**
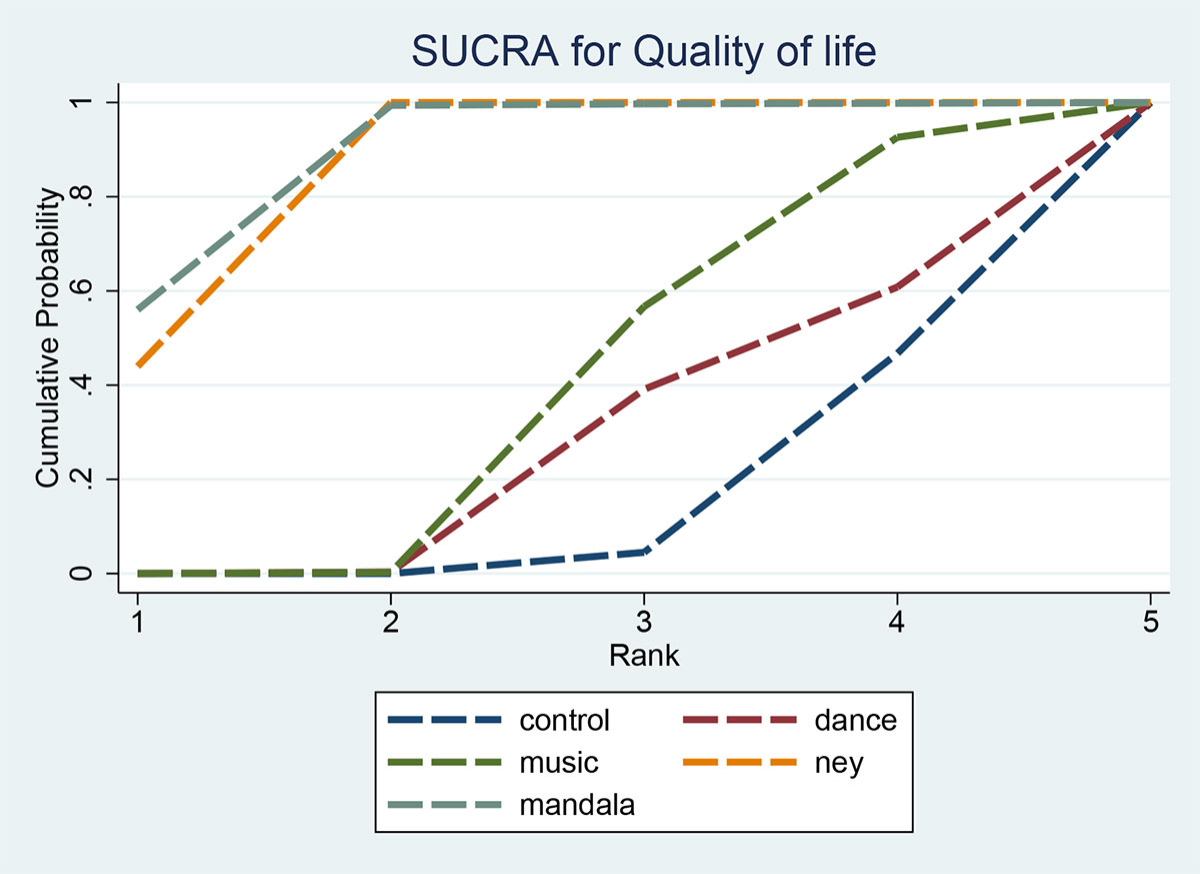
Area under the cumulative ranking curve for quality of life.

The league table indicated that, for improving the patients’ quality of life, mandala painting therapy outperformed routine care (SMD = 12.15, 95% CrI: 20.51, 3.76), dance therapy (SMD = 11.74, 95% CrI: 20.62, 2.92), and music therapy (SMD = 11.38, 95% CrI: 19.81, 2.91). Ney therapy was superior to routine care (SMD = 11.44, 95% CrI: 12.51, 10.36), dance therapy (SMD = 11.06, 95% CrI: 14.1, 8.0), and music therapy (SMD = 10.68, 95% CrI: 12.31, 9.04). Additional data are presented in **Table 4**. The interventions were ranked by SUCRA value as follows: mandala painting therapy (88.8%) > Ney therapy (85.9%) > music therapy (37.3%) > dance therapy (25.2%) > routine care (12.8%). The SUCRA results indicated that mandala painting therapy ranked high in improving quality of life (**Figure 6**).

**Table 4:** League table for quality of life.

**control**				
-0.39 (-3.24, 2.47)	**dance**			
-0.76 (-1.98, 0.46)	-0.38 (-3.47, 2.74)	**music**		
-11.44 (-12.51, -10.36)	-11.06 (-14.1, -8)	-10.68 (-12.31, -9.04)	**ney art**	
-12.15 (-20.51, -3.76)	-11.74 (-20.62, -2.92)	-11.38 (-19.81, -2.91)	-0.7 (-9.12, 7.75)	**mandala**

#### 3.5.4 Pain

**Figure 7:**
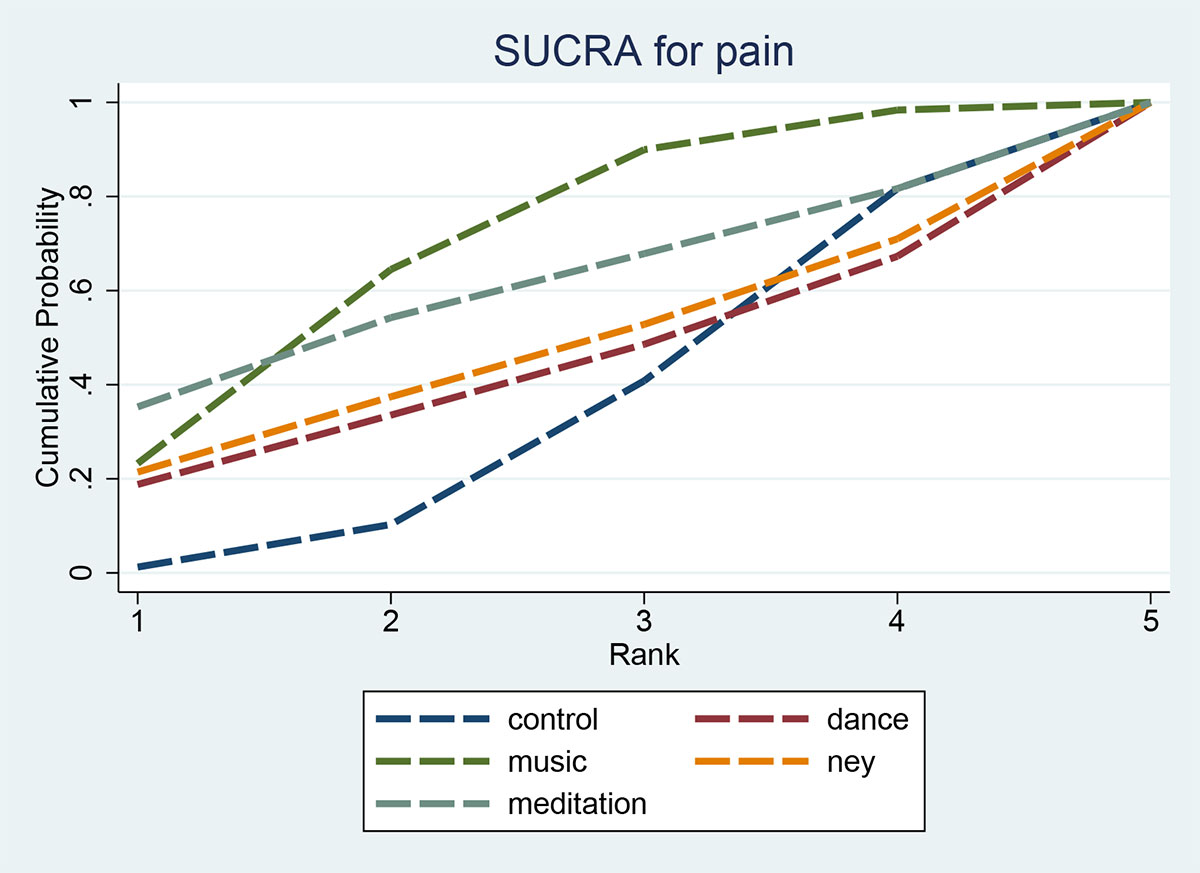
Area under the cumulative ranking curve for pain.

The league table showed no statistically significant differences between the interventions and control in the relief of pain. Additional data are presented in **Table 5**. The interventions were ranked by SUCRA value as follows: music therapy (69.0%) > meditation (59.8%) > Ney therapy (45.7%) > dance therapy (42.0%) > routine care (33.5%). Music therapy ranked first in the SUCRA ranking. However, since none of the pairwise comparisons showed statistically significant differences, this result merely indicated its potential for clinical application (**Figure 7**).

**Table 5:** League table for pain.

**control**				
0.67 (-38.64, 39.83)	**dance**			
9.09 (-5.64, 24.62)	8.46 (-33.15, 50.99)	**music**		
2.24 (-36.19, 41.04)	1.5 (-52.86, 56.74)	-6.85 (-48.33, 34.57)	**ney**	
8.36 (-33.28, 50.66)	7.73 (-49.17, 65.66)	-0.74 (-39.76, 38.71)	6.27 (-51.06, 62.87)	**meditation**

#### 3.5.5 Depression

**Figure 8:**
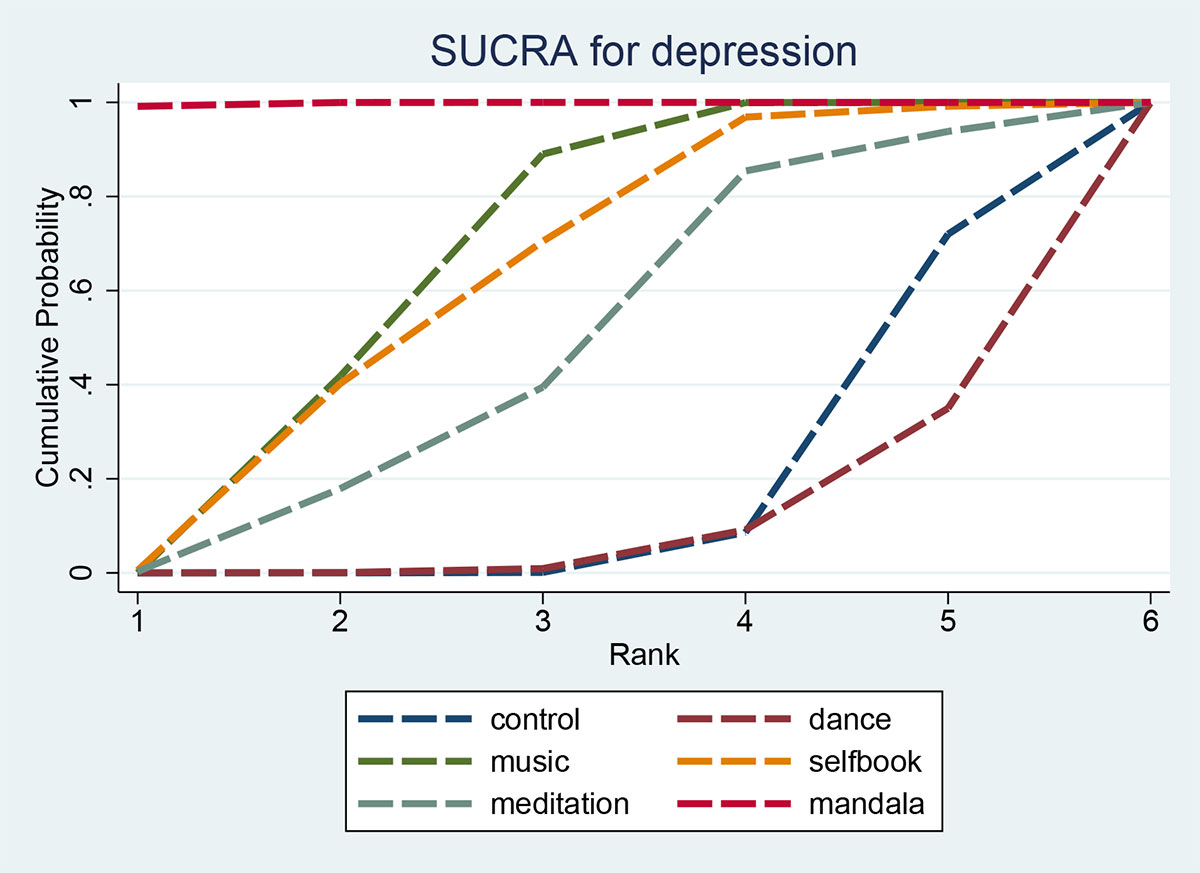
Area under the cumulative ranking curve for depression.

The league table indicated that, for reducing depressive symptoms among cancer survivors, music therapy demonstrated greater efficacy than routine care (SMD = -1.71, 95% CrI: -1.15, -2.26) and dance therapy (SMD = -2.0, 95% CrI: -0.64, -3.37). Self-book therapy also outperformed routine care (SMD = -1.70, 95% CrI: -0.12, -3.11). Mandala painting therapy demonstrated superior performance in all pairwise comparisons, exhibiting significantly greater efficacy than routine care (SMD = -3.95, 95% CrI: -2.93, -4.97), dance therapy (SMD = -4.25, 95% CrI: -2.65, -5.87), music therapy (SMD = -2.25, 95% CrI: -1.09, -3.4), self-book therapy (SMD = -2.35, 95% CrI: -0.54, -4.16), and meditation therapy (SMD = -2.85, 95% CrI: -0.8, -4.88). Additional data are presented in **Table 6**. The interventions were ranked by SUCRA value as follows: mandala painting therapy (99.8%) > music therapy (66.2%) > meditation (61.5%) > self-book therapy (47.3%) > dance therapy (9.0%) > routine care (16.1%). These results suggested that mandala painting therapy may be a superior intervention for alleviating depressive symptoms in cancer survivors (**Figure 8**).

**Table 6:** League table for depression.

**control**					
-0.3 (-1.56, 0.95)	**dance**				
1.71 (1.15, 2.26)	2 (0.65, 3.37)	**music**			
1.6 (0.12, 3.11)	1.9 (-0.05, 3.86)	-0.1 (-1.69, 1.51)	**selfbook**		
1.11 (-0.65, 2.87)	1.4 (-0.74, 3.57)	-0.6 (-2.27, 1.08)	-0.5 (-2.8, 1.81)	**meditation**	
3.95 (2.93, 4.97)	4.25 (2.65, 5.87)	2.25 (1.09, 3.4)	2.35 (0.54, 4.16)	2.85 (0.8, 4.88)	**mandala**

### 3.6 Results of heterogeneity, sensitivity, and meta-regression analyses

The comparison between music therapy and routine care revealed high heterogeneity in anxiety (I^2^ = 96.9%), pain (I^2^ = 94.5%), and quality of life (I^2^ = 80.6%) outcomes (**Supplementary Figure 1**-**S13**), with low heterogeneity in other outcomes. Since no closed loops were formed among the five outcomes included in this study for any intervention, it was impossible to perform an inconsistency test. Given the high heterogeneity observed in the outcomes of anxiety, pain, and quality of life, sensitivity analyses and meta-regression analyses were conducted. Following the exclusion of studies reporting incomplete data, the heterogeneity in anxiety and quality of life outcomes decreased (I^2^ = 66.7% and 80.5%, respectively), but remained high. For the outcome of pain, sensitivity analysis was not conducted because the original network became incomplete after the exclusion, making it impossible to perform valid network comparisons. Overall, after the exclusion, the effect direction, 95% CrI of pooled effect sizes, and SUCRA ranking did not have substantial changes for the outcomes of anxiety and quality of life. Additionally, univariate meta-regression analyses were conducted using the publication year, sample size, and gender ratio as covariates. The results showed that the 95% confidence intervals (CI) for the regression coefficients (B) of all covariates contained 0, suggesting no statistical association between these covariates and the effect size. The analysis indicated that publication year, sample size, and gender ratio may not be the primary sources of heterogeneity.

### 3.7 Publication bias

**Figure 9:**
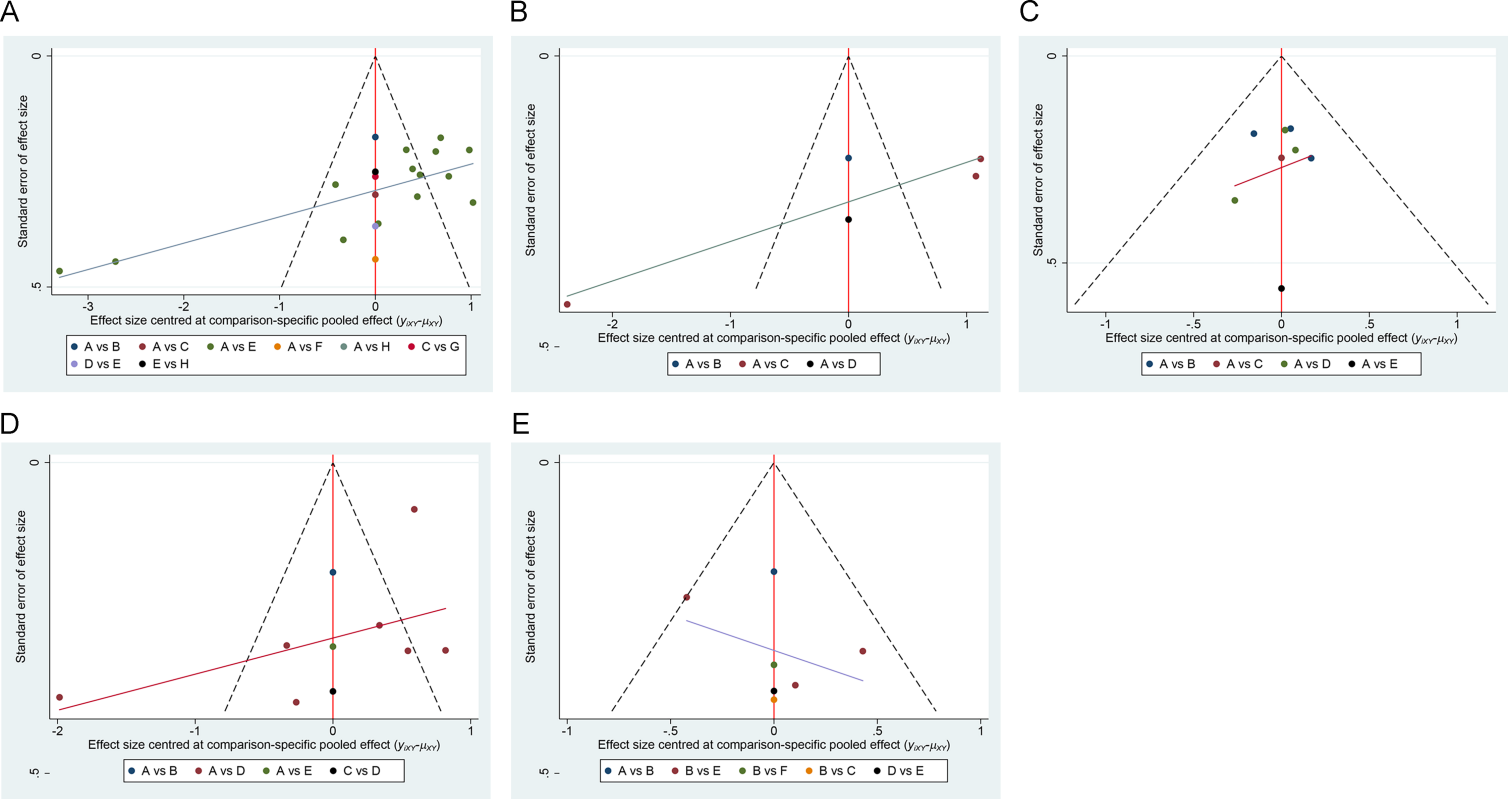
Funnel plots for risk of bias assessment: anxiety **(A)**, fatigue **(B)**, quality of life **(C)**, pain **(D)**, depression **(E)**.

The comparison-adjusted funnel plots (**Figure 9**) showed a certain degree of asymmetry in the scatter plots for the outcomes of anxiety (**Figure 9A**) and pain (**Figure 9D**). Meanwhile, the results of Egger’s test were statistically significant (P = 0.0048 and P = 0.0457, respectively), suggesting that publication bias or small-sample effects may be present for these outcomes. For the outcomes of fatigue, depression, and quality of life, due to the small number of included studies (**Figures 9B, C, E**), the funnel plots showed a limited number of scattered points and failed to form a stable distribution pattern. Furthermore, the prerequisites for the application of Egger’s test were not met. Therefore, no further quantitative tests were conducted. Overall, some outcome measures in this study may be subject to a certain risk of publication bias, and such bias may influence the estimated effect sizes. Therefore, the study results should be interpreted with caution in the context of clinical practice.

## 4 Discussion

This study is the first to systematically evaluate the effectiveness of various art therapies for anxiety, depression, pain, fatigue, and quality of life among cancer survivors. The NMA revealed a positive effect of art therapy on some psychological outcomes and the quality of life, consistent with the findings of previous studies (14, 54, 57, 58). From a mechanistic perspective, according to the gate control theory (59), musical stimuli can interfere with the transmission of pain signals. At the neurochemical level, pleasant music can induce the release of endogenous opioid peptides and dopamine, activate the brain’s reward system, and elevate the pain threshold (60). Meanwhile, music may also alleviate an individual’s stress response and psychological distress from a neuroimmunological perspective (61) by modulating the hypothalamic-pituitary-adrenal (HPA) axis and reducing cortisol levels (62). Mandala painting therapy can guide individuals into a “flow” state through structured symmetrical patterns, downregulate the excessive activation of the default-mode network (DMN), thereby reducing rumination associated with depression (63) and promoting emotional regulation (64). Active creation also helps enhance self-efficacy and alleviate learned helplessness (65). These findings suggest that different art therapies may exert therapeutic effects on cancer survivors through distinct psychoneurobiological pathways.

In this study, the outcomes of anxiety, pain, and quality of life exhibited high heterogeneity, suggesting that the effectiveness of art therapy may be jointly influenced by multiple factors. Compared to pharmacological interventions, art therapy is inherently highly individualized and context-dependent. Significant differences are found across studies in terms of intervention form, administration frequency, duration, setting, and the professional background of practitioners. Furthermore, the psychological processing pathways activated by different art media are not entirely consistent, which may also lead to inconsistent effectiveness across different outcome measures. Regarding pain, the subjective nature of pain perception and its susceptibility to multiple factors, such as cancer type, disease stage, analgesic regimens, and individual psychological states, may further increase heterogeneity among studies. Moreover, insufficient reporting of cancer staging, baseline treatments, and patient clinical characteristics in some studies, along with measurement discrepancies arising from the use of different scale instruments, may further contribute to clinical heterogeneity across studies. Nevertheless, following sensitivity analyses, the overall effect direction and the SUCRA ranking did not change significantly, suggesting that the results of the main analysis remained relatively robust. Furthermore, we observed that in the 28 included studies, approximately 64% of art interventions were administered by trained healthcare professionals, suggesting that a healthcare professional-led model appears to be feasible and has the potential for widespread adoption for integrating art therapy into routine clinical practice. Given its non-invasiveness, relatively low costs, and high patient acceptability (66), art therapy has the potential to serve as an important adjunct to comprehensive supportive care for cancer survivors.

The included studies spanned diverse cultural contexts, with more than half originating from non-Western countries. Individuals from different cultural backgrounds may differ in their perception, expression, and coping styles regarding psychological states and somatic symptoms. This cultural heterogeneity may, to some extent, influence the effectiveness of art therapy interventions. Previous studies have shown that in Western cultural contexts, music is more frequently viewed as a “psychological placebo” for alleviating acute psychological stress. Its effects primarily manifest as immediate emotional improvement and relief from subjective distress, while its efficacy in alleviating fatigue and physical pain may be relatively limited (67). In contrast, patients from Eastern cultural backgrounds tend to express psychological distress through somatization (68) and may interpret the experience of fatigue as a depletion of “Qi” or vital energy. Culturally specific musical forms, such as Sufi music centered on the Ney flute, may further enhance psychological comfort and spiritual support by evoking deeper emotional resonance and cultural identity, thereby exerting a more pronounced effect on outcomes such as fatigue (15). Future research should focus more on cultural adaptability to optimize therapeutic efficacy.

Although this study provides the first systematic framework for comparing the efficacy of various art therapies, several limitations exist that require cautious interpretation. First, the search does not include gray literature or non-English publications, and the evidence is highly concentrated in Asia and North America, which may affect the global generalizability of the findings. Second, differences in patient clinical characteristics, measurement tools, intervention protocols, and assessment time points across studies may lead to high heterogeneity. Some original trials fail to explicitly report cancer staging or baseline treatment regimens. The evidence network exhibits a star-shaped structure, suggesting that current effect sizes may only reflect averaged effects. Although SMDs were used to synthesize data from different scales, differences in symptom sensitivity and weighting of somatization symptoms across scales may also contribute to heterogeneity. Furthermore, the lack of standardization in interventions and the wide range of assessment time points may have diluted short-term benefits. Therefore, future studies should establish standardized intervention and assessment frameworks, clearly define measurement time points for both the acute and maintenance phases, and provide more reliable evidence-based grounds for comparing different art therapies.

## 5 Conclusion

Art interventions are gaining increasing attention in cancer supportive care. This study provides preliminary evidence-based findings through an NMA, which indicates that various art therapies may offer potential benefits in improving the psychological well-being and reducing the symptom burden of cancer survivors. However, given the star-shaped structure of the existing evidence network and the presence of clinical heterogeneity, the cumulative probability ranking should be interpreted as exploratory guidance rather than definitive conclusions. Additional high-quality, standardized, multi-arm head-to-head RCTs are required in the future to further validate the efficacy of art therapy. In clinical practice, healthcare professionals should take into account patients’ cultural backgrounds, personal preferences, and disease stage to carefully design individualized art intervention plans, thereby maximizing benefits for patients’ physical and mental well-being.

## Data Availability

The original contributions presented in the study are included in the article/**Supplementary Material**. Further inquiries can be directed to the corresponding authors.
