# Supplementary material for: Effects of different art therapies on the psychological well-being and quality of life in cancer survivors: a systematic review and network meta-analysis
Source: Front Psychiatry. 2026 Jul 6;17:1817092. doi: 10.3389/fpsyt.2026.1817092 (PMC13383102; doi:10.3389/fpsyt.2026.1817092)
Supplement: Supplementary file 1 [file SupplementaryFile1.docx]

Supplementary Material

**Supplementary Table S1: Search Strategy**

The search was conducted in September 2025. Below is a summary of the search strategy executed in the aforementioned databases.

1) Pubmed

2) Embase

3) Cochrane

4) Web of Science

| Pubmed | |
| --- | --- |
| # | Query |
| 1 | Neoplasms[MeSH Terms] |
| 2 | '’Cancer*''[Title/Abstract] OR ''Malignanc*''[Title/Abstract] OR ''malignant neoplastic disease''[Title/Abstract] OR ''malignant  tumour''[Title/Abstract] OR ''Neoplasia*''[Title/Abstract] OR ''Neoplasm*''[Title/Abstract] OR ''Tumor*[Title/Abstract] |
| 3 | #1 OR #2 |
| 4 | ((((((Art Therapy[MeSH Terms]) OR (Art[MeSH Terms])) OR (Music  Therapy[MeSH Terms])) OR (Music[MeSH Terms])) OR (Horticultural  Therapy[MeSH Terms])) OR (Paintings[MeSH Terms])) OR (Sensory Art Therapies[MeSH Terms]) |
| 5 | ''art''[Title/Abstract] OR ''arts''[Title/Abstract] OR  ''caricatures''[Title/Abstract] OR ''cartoon*''[Title/Abstract] OR ''cleopatra needle''[Title/Abstract] OR ''comic books as  topic''[Title/Abstract] OR ''drawing*''[Title/Abstract] OR ''Garden* Therapy''[Title/Abstract] OR ''graphic novels as  topic''[Title/Abstract] OR ''Horticultur* Therapy''[Title/Abstract] OR ''melotherapy''[Title/Abstract] OR ''music''[Title/Abstract] OR  ''musical test''[Title/Abstract] OR ''orchestra''[Title/Abstract] OR ''painting*''[Title/Abstract] OR ''philately''[Title/Abstract] OR  ''pictorial works as topic''[Title/Abstract] OR  ''portrait*''[Title/Abstract] OR ''sculpture''[Title/Abstract] OR ''Song*''[Title/Abstract] OR ''Therapeutic  Horticulture''[Title/Abstract] OR ''Vocal Melod*''[Title/Abstract] |
| 6 | #4 OR #5 |
| 7 | random*[Title/Abstract] |
| 8 | #3 AND #6 AND #7 |

| Embase | |
| --- | --- |
| # | Query |
| 2 | 'malignant neoplasm'/exp |
| 4 | 'cancer*':ti,ab,kw OR 'malignanc*':ti,ab,kw OR 'malignant neoplastic disease':ti,ab,kw OR 'malignant tumour':ti,ab,kw OR  'neoplasia*':ti,ab,kw OR 'neoplasm*':ti,ab,kw OR 'tumor*':ti,ab,kw |
| 5 | #2 OR #4 |
| 7 | 'art therapy'/exp OR 'art'/exp OR 'music therapy'/exp OR 'music'/exp OR 'horticultural therapy'/exp OR 'painting'/exp OR 'drawing'/exp |
| 9 | 'art':ti,ab,kw OR 'arts':ti,ab,kw OR 'caricatures':ti,ab,kw OR  'cartoon*':ti,ab,kw OR 'cleopatra needle':ti,ab,kw OR 'comic books as  topic':ti,ab,kw OR 'drawing*':ti,ab,kw OR 'garden* therapy':ti,ab,kw OR 'graphic novels as topic':ti,ab,kw OR 'horticultur* therapy':ti,ab,kw  OR 'melotherapy':ti,ab,kw OR 'music':ti,ab,kw OR 'musical  test':ti,ab,kw OR 'orchestra':ti,ab,kw OR 'painting*':ti,ab,kw OR  'philately':ti,ab,kw OR 'pictorial works as topic':ti,ab,kw OR  'portrait*':ti,ab,kw OR 'sculpture':ti,ab,kw OR 'song*':ti,ab,kw OR 'therapeutic horticulture':ti,ab,kw OR 'vocal melod*':ti,ab,kw |
| 10 | #7 OR #9 |
| 11 | 'random*':ti,ab,kw |
| 12 | #5 AND #10 AND #11 |

| Cochrane Library | |
| --- | --- |
| # | Query |
| 1 | MeSH descriptor: [Neoplasms] explode all trees |
| 2 | ('Cancer*' OR 'Malignanc*' OR 'malignant neoplastic disease' OR  'malignant tumour' OR 'Neoplasia*' OR 'Neoplasm*' OR 'Tumor*):ti,ab,kw |

| 3 | #1 OR #2 |
| --- | --- |
| 4 | MeSH descriptor: [Art Therapy] explode all trees |
| 5 | MeSH descriptor: [Art] explode all trees |
| 6 | MeSH descriptor: [Music Therapy] explode all trees |
| 7 | MeSH descriptor: [Music] explode all trees |
| 8 | MeSH descriptor: [Horticultural Therapy] explode all trees |
| 9 | MeSH descriptor: [Paintings] explode all trees |
| 10 | MeSH descriptor: [Art Therapy] explode all trees |
| 11 | MeSH descriptor: [Drawing] explode all trees |
| 12 | ('art' OR 'arts' OR 'caricatures' OR 'cartoon*' OR 'cleopatra needle'  OR 'comic books as topic' OR 'drawing*' OR 'Garden* Therapy' OR  'graphic novels as topic' OR 'Horticultur* Therapy' OR 'melotherapy' OR 'music' OR 'musical test' OR 'orchestra' OR 'painting*' OR 'philately' OR 'pictorial works as topic' OR 'portrait*' OR 'sculpture' OR 'Song*' OR 'Therapeutic Horticulture' OR 'Vocal Melod*'):ti,ab,kw |
| 13 | #4 OR #5 OR #6 OR #7 OR #8 OR #9 OR #10 OR #11 OR #12 |
| 14 | (random*):ti,ab,kw |
| 15 | #3 AND #13 AND #14 |

| Web of Science | |
| --- | --- |
| # | Query |
| 1 | TS=((Cancer*) OR (Malignanc*) OR (malignant neoplastic disease)  OR (malignant tumour) OR (Neoplasia*) OR (Neoplasm*) OR (Tumor*)) |
| 2 | TS=((art) OR (arts) OR (caricatures) OR (cartoon*) OR (cleopatra needle) OR (comic books as topic) OR (drawing*) OR (Garden* |

|  | Therapy) OR (graphic novels as topic) OR (Horticultur* Therapy) OR (melotherapy) OR (music) OR (musical test) OR (orchestra) OR (painting*) OR (philately) OR (pictorial works as topic) OR  (portrait*) OR (sculpture) OR (Song*) OR (Therapeutic Horticulture) OR (Vocal Melod*)) |
| --- | --- |
| 3 | TS=(Random*) |
| 4 | #3 AND #2 AND #1 |


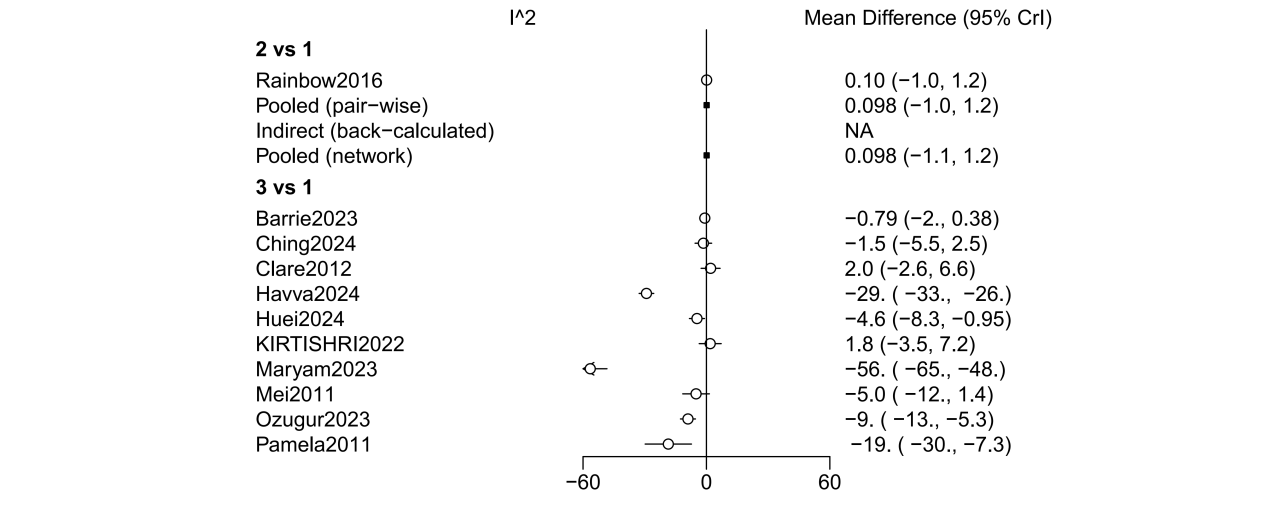


**Supplementary Figure S1：Heterogeneity assessment results for anxiety outcomes: Group coding 1 = control; 2 = dance; 3 = music**


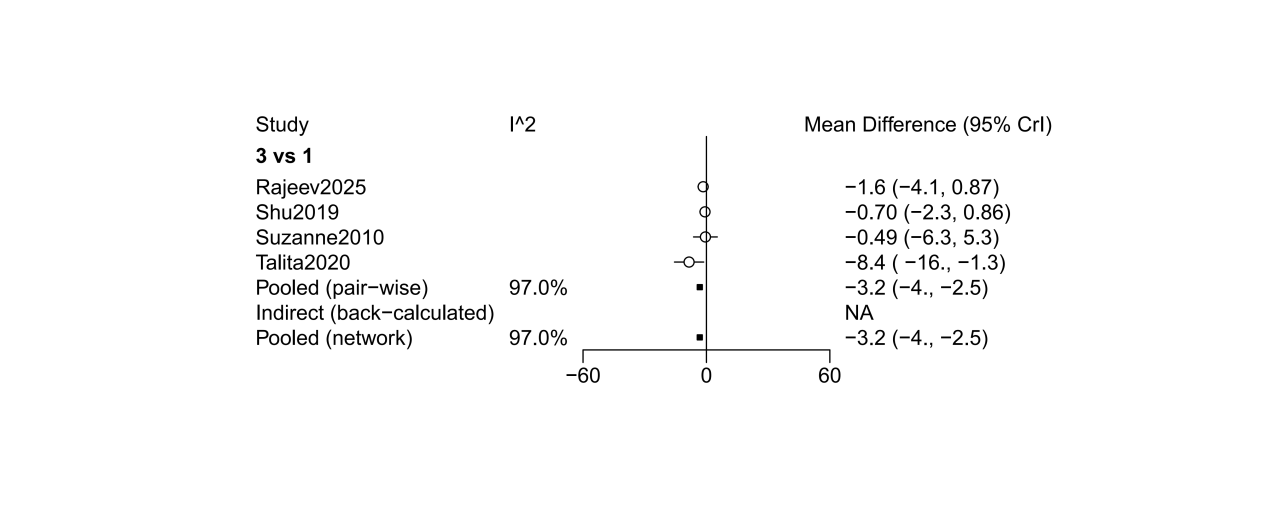


**Supplementary Figure S2：Heterogeneity assessment results for anxiety outcomes: Group coding 1 = control; 3 = music**


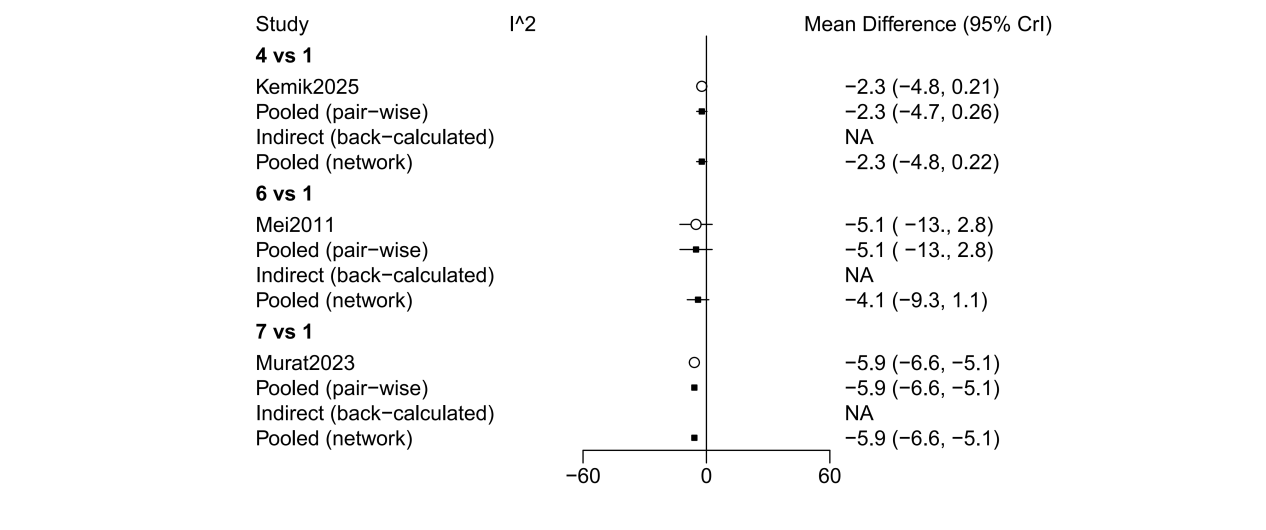


**Supplementary Figure S3：Heterogeneity assessment results for anxiety outcomes: Group coding 1 = control; 4 = mandala; 6 = verbal; 7 = ney**


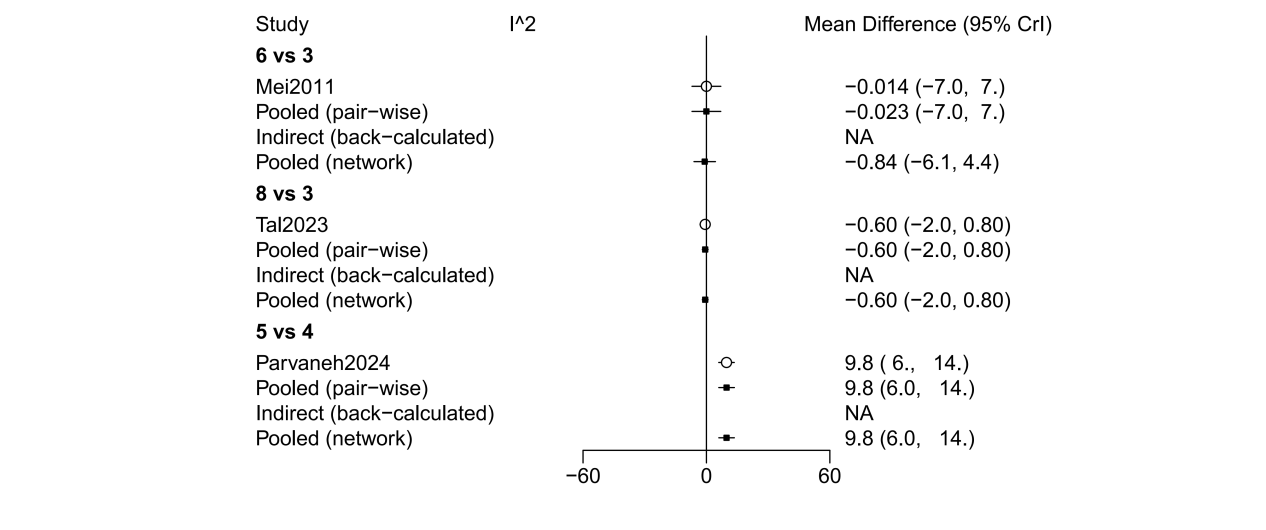


**Supplementary Figure S4：Heterogeneity assessment results for anxiety outcomes: Group coding 6 = verbal; 3 = music; 8 = meditation; 5 = sudoku; 4 = mandala**


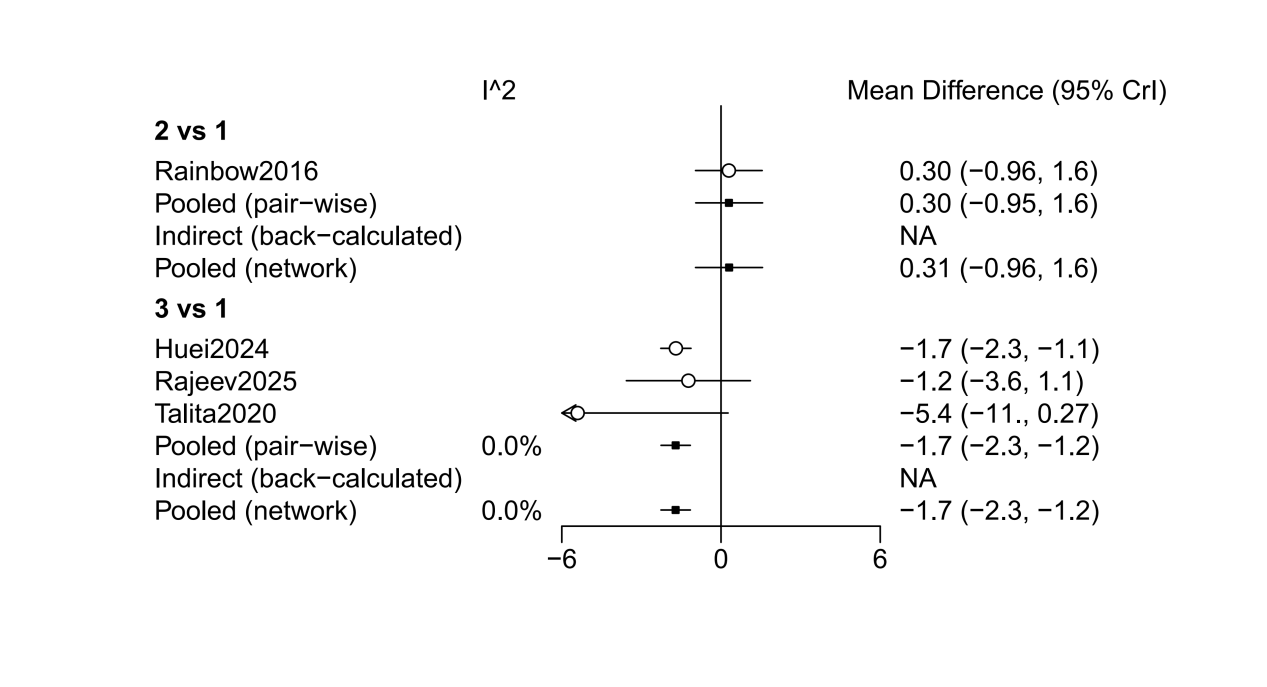


**Supplementary Figure S5：Heterogeneity assessment results for depression outcomes: Group coding 1 = control; 2 = dance; 3 = music**


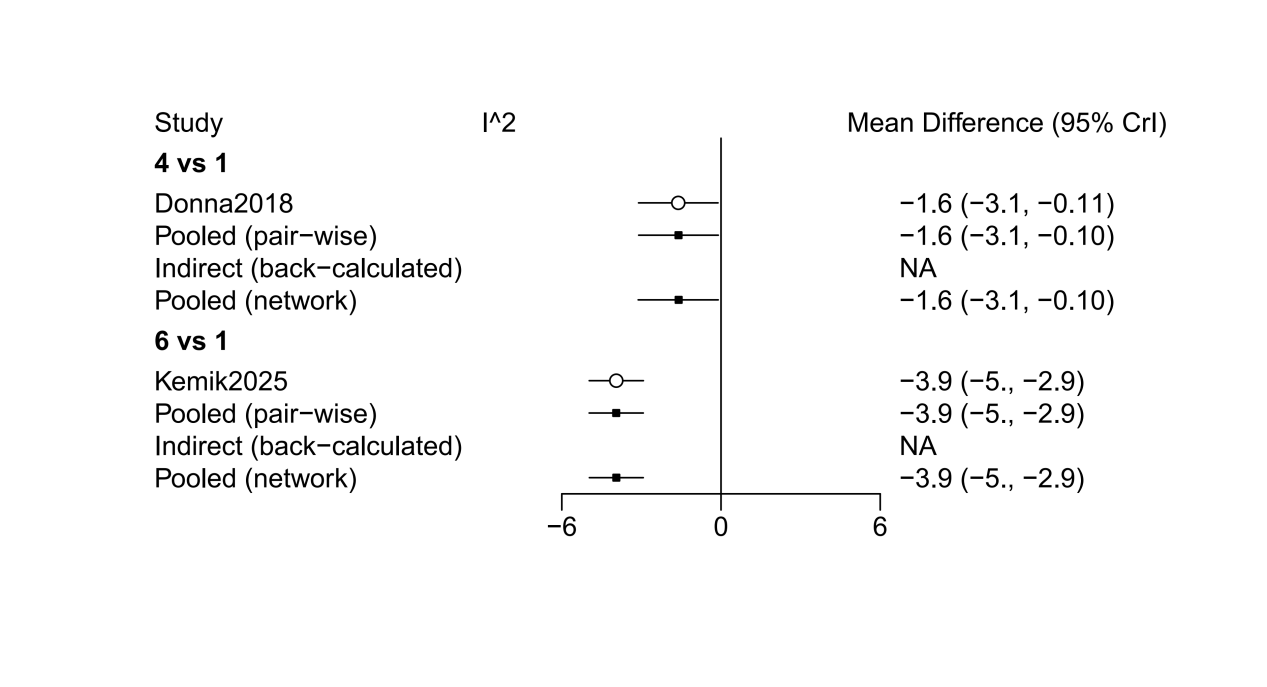


**Supplementary Figure S6：Heterogeneity assessment results for depression outcomes: Group coding 1 = control; 4 = selfbook; 6 = mandala**


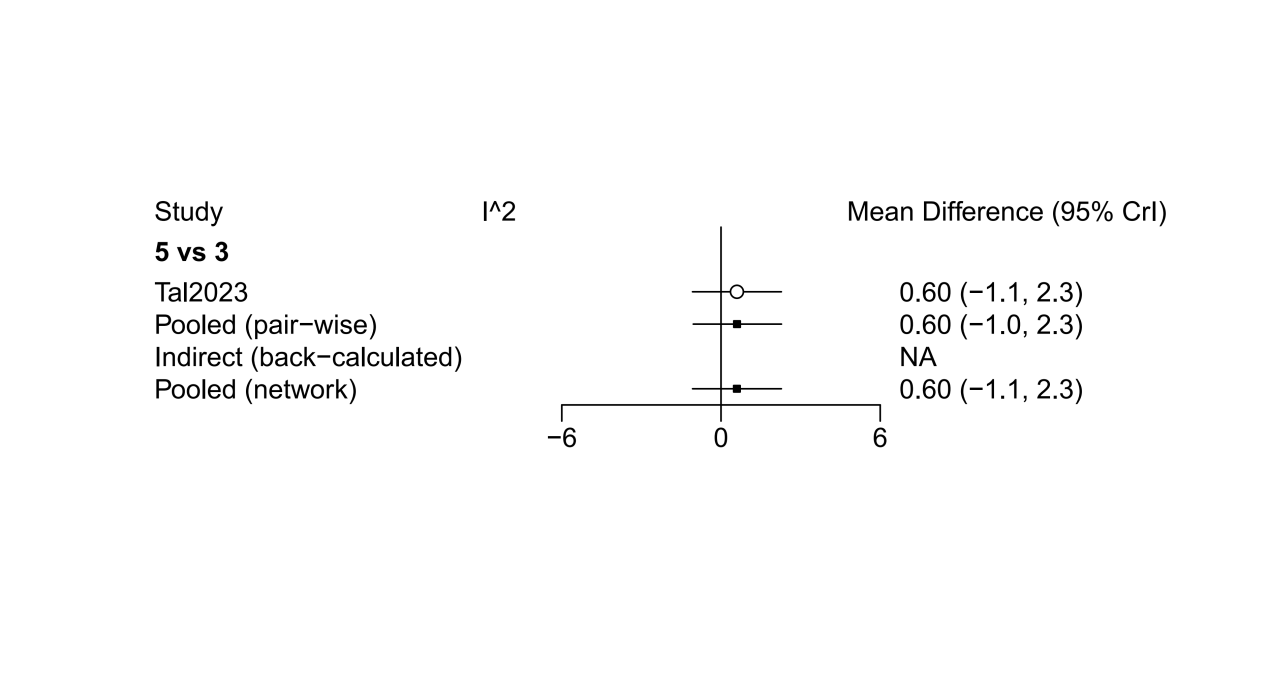


**Supplementary Figure S7：Heterogeneity assessment results for depression outcomes: Group coding 3 = music; 5 = meditation**


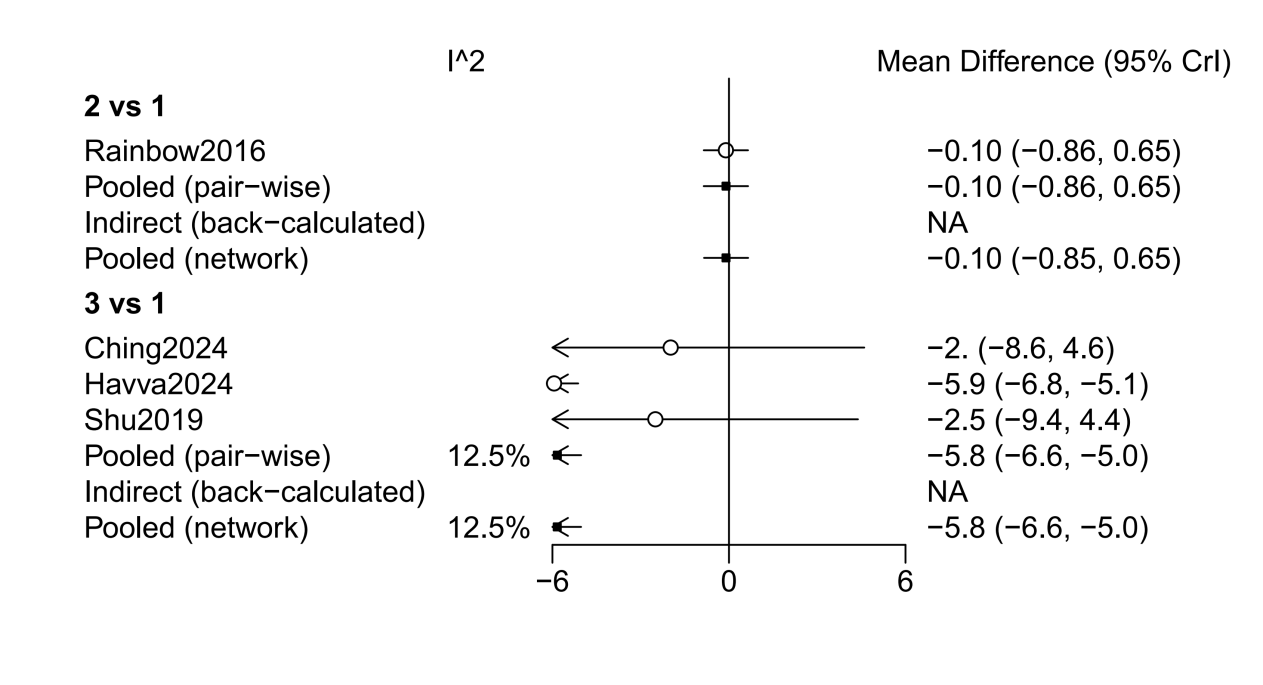


**Supplementary Figure S8：Heterogeneity assessment results for fatigue outcomes: Group coding 1 = control; 2 = dance; 3 = music**


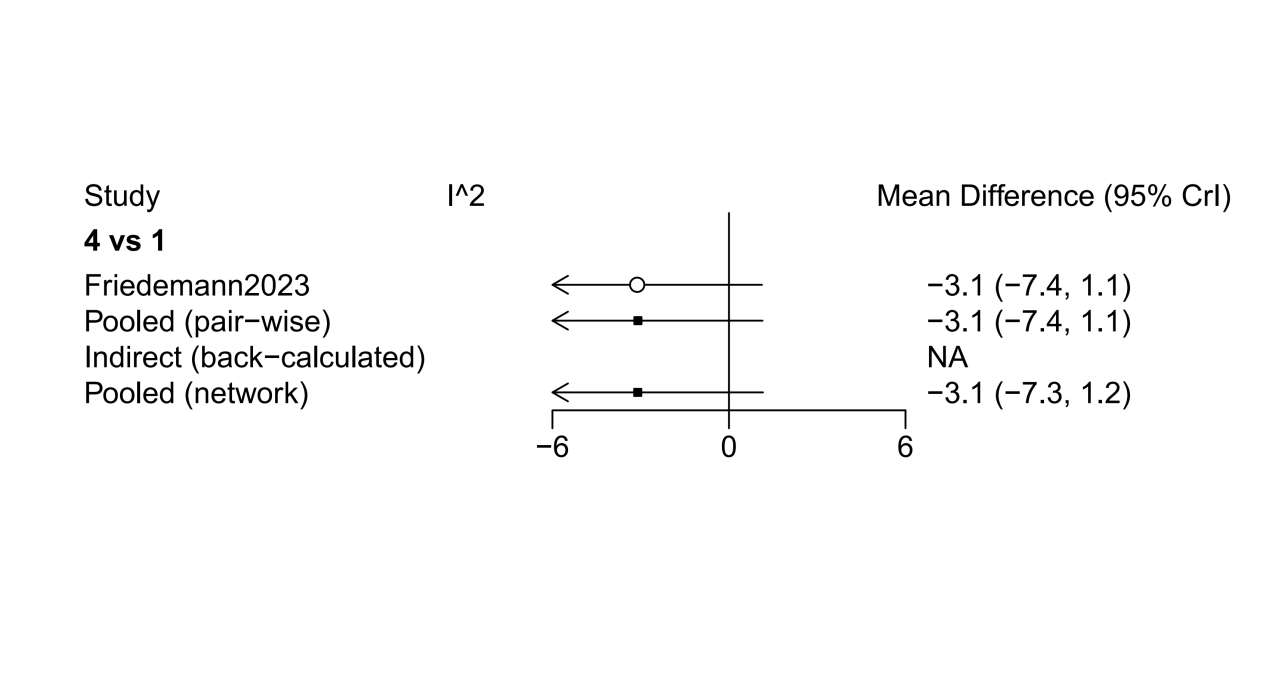


**Supplementary Figure S9：Heterogeneity assessment results for fatigue outcomes: Group coding 1 = control; 4 = tango**


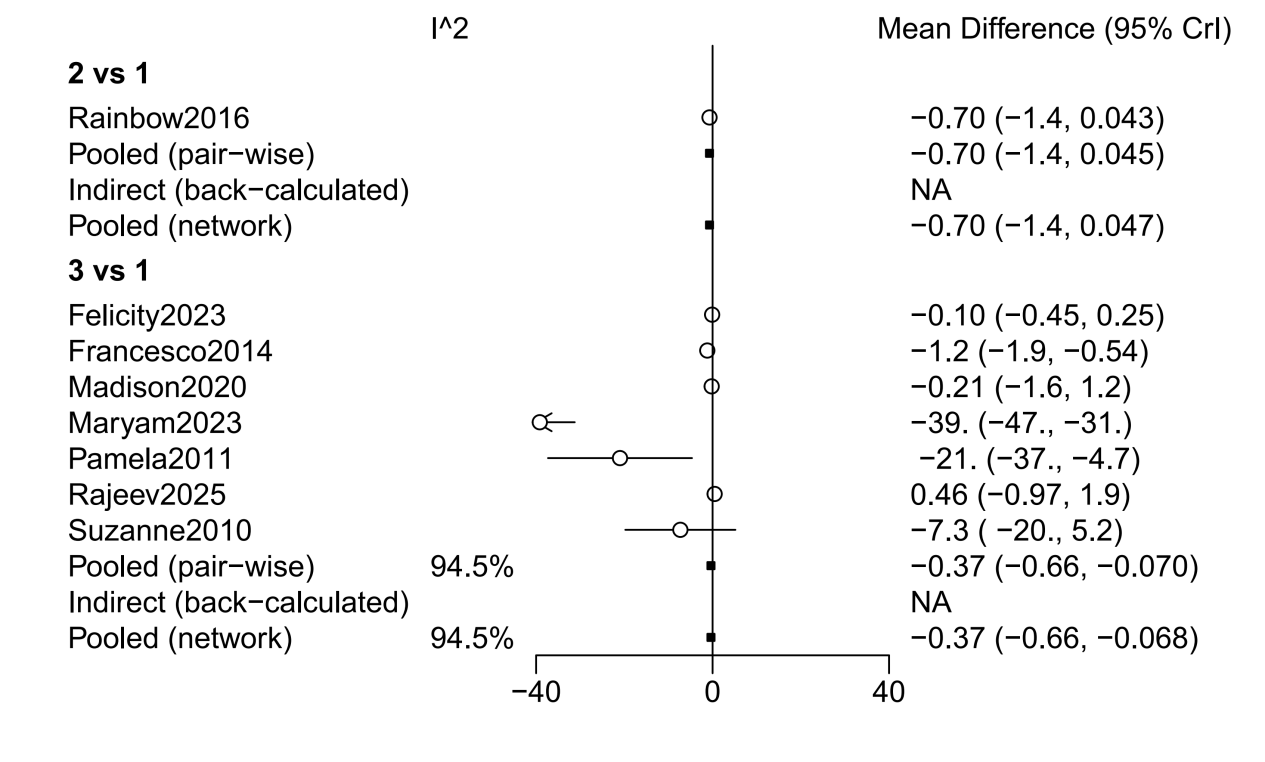


**Supplementary Figure S10：Heterogeneity assessment results for pain outcomes: Group coding 1 = control; 2 = dance; 3 = music**


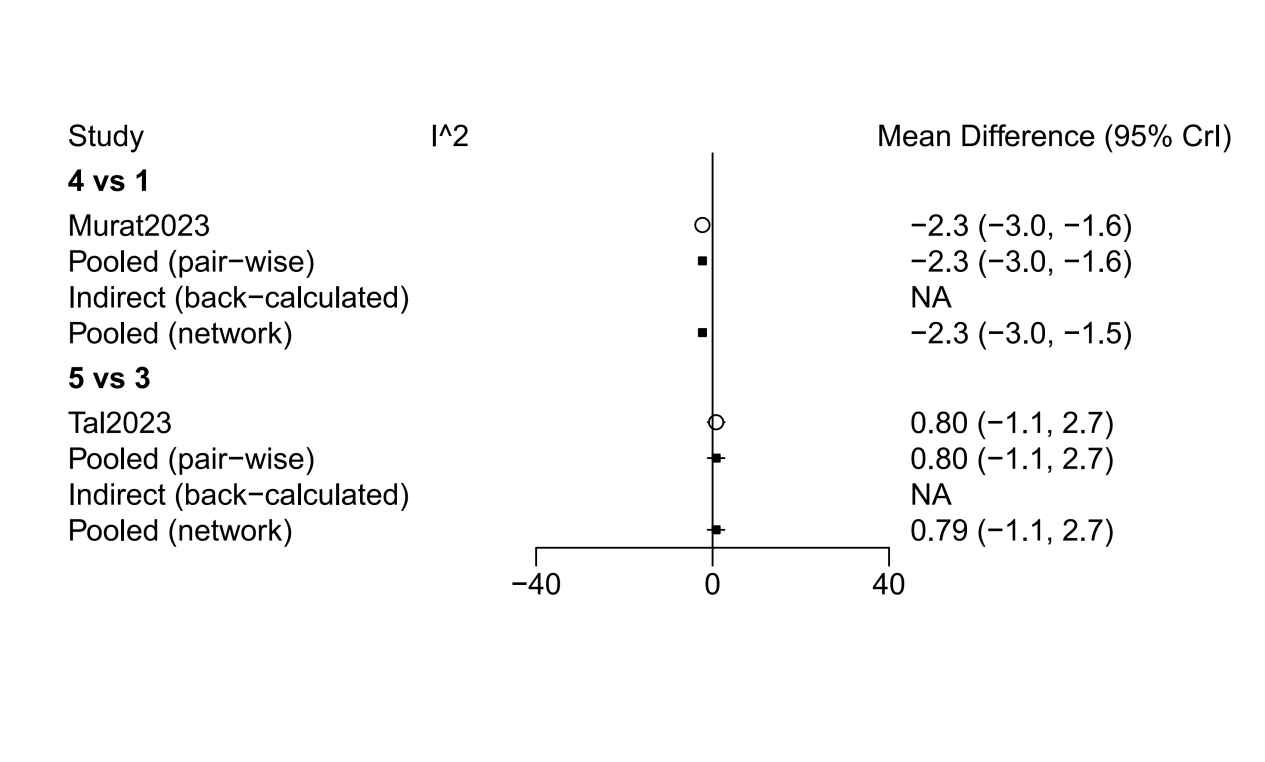


**Supplementary Figure S11：Heterogeneity assessment results for pain outcomes: Group coding 1 = control; 4 = ney; 5 = meditation**


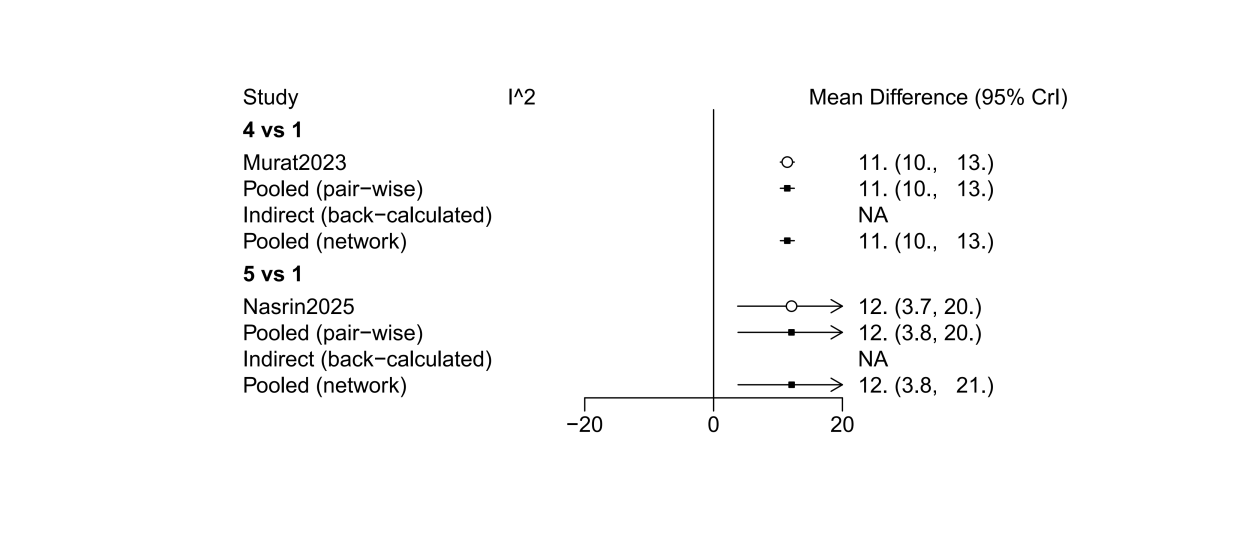


**Supplementary Figure S12：Heterogeneity assessment results for quality of life outcomes: Group coding 1 = control; 4 = ney; 5 = meditation**


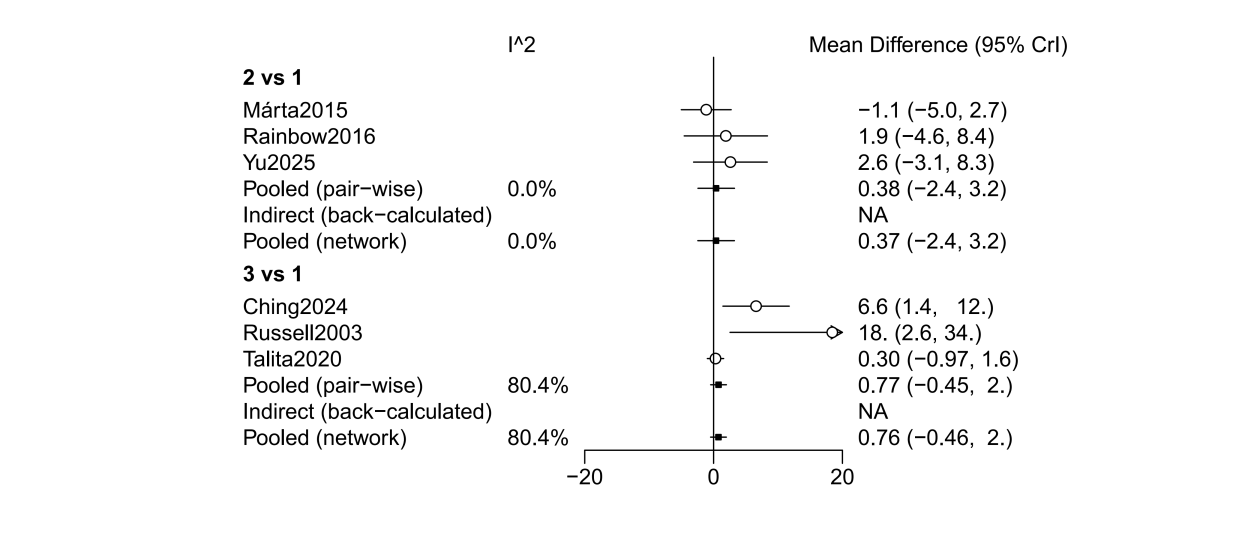


**Supplementary Figure S13：Heterogeneity assessment results for quality of life outcomes: Group coding 1 = control; 2 = dance; 3 = music**
